# The Essential Role of Angiogenesis in Adenosine 2A Receptor Deficiency-mediated Impairment of Wound Healing Involving c-Ski via the ERK/CREB Pathways

**DOI:** 10.7150/ijbs.98856

**Published:** 2024-08-19

**Authors:** Yan Peng, Renping Xiong, Bo Wang, Xing Chen, Yalei Ning, Yan Zhao, Nan Yang, Jing Zhang, Changhong Li, Yuanguo Zhou, Ping Li

**Affiliations:** Department of Army Occupational Disease, State Key Laboratory of Trauma, Burn and Combined Injury, Daping Hospital, Army Medical University (Third Military Medical University), 10 Changjiang Zhilu, Chongqing 400042, People's Republic of China.

**Keywords:** Wound healing, A_2A_R, Angiogenesis, c-Ski, Endothelial cell

## Abstract

Adenosine receptor-mediated signaling, especially adenosine A_2A_ receptor (A_2A_R) signaling, has been implicated in wound healing. However, the role of endothelial cells (ECs) in A_2A_R-mediated wound healing and the mechanism underlying this effect are still unclear. Here, we showed that the expression of A_2A_R substantially increased after wounding and was especially prominent in granulation tissue. The delaying effects of A_2A_R knockout (KO) on wound healing are due mainly to the effect of A_2A_R on endothelial cells, as shown with A_2A_R-KO and EC-A_2A_R-KO mice. Moreover, the expression of c-Ski, which is especially prominent in CD31-positive cells in granulation tissue, increased after wounding and was decreased by both EC-A_2A_R KO and A_2A_R KO. In human microvascular ECs (HMECs), A_2A_R activation induced EC proliferation, migration, tubule formation and c-Ski expression, whereas c-Ski depletion by RNAi abolished these effects. Mechanistically, A_2A_R activation promotes the expression of c-Ski through an ERK/CREB-dependent pathway. Thus, A_2A_R-mediated angiogenesis plays a critical role in wound healing, and c-Ski is involved mainly in the regulation of angiogenesis by A_2A_R via the ERK/CREB pathway. These findings identify A_2A_R as a therapeutic target in wound repair and other angiogenesis-dependent tissue repair processes.

## Introduction

The enhancement of wound healing has been a goal of medical practitioners for thousands of years. Therapeutic interventions that activate the innate repair mechanism of native tissue and promote healing are of particular interest. Adenosine, a potent endogenous physiological mediator, and its receptors play key roles in tissue repair and wound healing^1^-^4^. Moreover, caffeine, a major component of many beverages, such as coffee and tea, is an adenosine receptor antagonist and has inhibitory effects on tissue repair and wound healing^5^-^7^. Therefore, exploration of the mechanisms of adenosine and its receptors in wound repair is very important.

The adenosine A_2A_ receptor (A_2A_R), a subtype of G protein-coupled adenosine receptor, is broadly expressed on most cell types involved in wound healing, including macrophages, fibroblasts and microvascular endothelial cells (ECs)^8^. We and others have demonstrated that the topical application of A_2A_R agonists promotes healing of normal wounds^9^-^11^ and diabetic wounds^8^, ^12^, ^13^, and one such agonist, sonedenoson, is currently being evaluated as a therapy for diabetic foot ulcers^8^, ^11^. In addition, polydeoxyribonucleotides have been used to improve wound healing in clinical studies through A_2A_R activation^14^. Although A_2A_R activation reportedly accelerates the wound healing process by affecting ECs and stimulating angiogenesis^15^, relieving inflammation^15^, ^16^, and affecting fibroblasts and epithelial cells^13^, ^17^, ^18^, the roles of these regulatory effects in A_2A_R-mediated healing have not been fully elucidated. Specifically, the regulation of ECs in A_2A_R-mediated wound healing and the mechanism underlying this effect are still unclear.

The cellular Sloan-Kettering Institute (c-Ski) is an intracellular homolog of the virus oncogene *v-ski* and is involved in various physiological and pathological processes, such as the proliferation of hematopoietic cells, muscle regeneration, bone and nervous system development, synaptic projection, and tumorigenesis^19^-^23^. Further research has shown that c-Ski is involved in the repair of injured skeletal muscle, liver and other tissues^24^, ^25^. In recent years, we demonstrated that c-Ski is a wound repair factor that can accelerate wound healing in skin tissue and the brain^26^, ^27^. These effects are related to c-Ski's role as a multifunctional transcription regulator^28^, ^29^ that is involved in cell proliferation, transformation, secretion of the extracellular matrix and the inflammatory response. In particular, c-Ski is expressed on ECs^30^, and its overexpression promotes the proliferation of ECs^31^, ^32^. However, whether A_2A_R regulates wound healing through c-Ski's effects on ECs is unclear.

The extracellular signal-regulated kinase (ERK)/cAMP response element binding protein (CREB) pathway, an upstream signal, can transform extracellular stimulation into intracellular responses to promote the proliferation and differentiation of cells^33^. Moreover, the ERK/CREB pathway has a regulatory effect on angiogenesis^34^-^36^ and is also the canonical pathway of A_2A_R regulation^37^-^39^. Additionally, it has been reported that the expression of c-Ski can be regulated by the ERK/CREB pathway^40^. Thus, we speculated that A_2A_R may regulate c-Ski expression through the ERK/CREB pathway.

In this study, we explored the effect and mechanism of A_2A_R in regulating skin wound healing and its relationship with c-Ski. First, a full-thickness excisional wound model in mice was used to observe the expression of A_2A_R and explore its role in angiogenic regulation during wound healing via genetic [A_2A_R knockout (A_2A_R KO) and EC-specific A_2A_R knockout (EC-A_2A_R KO) mice] blockade of A_2A_R. Furthermore, we examined the effects of A_2A_R on the expression of c-Ski to explore its role in A_2A_R-mediated angiogenesis and wound healing by using A_2A_R KO and EC-A_2A_R KO mice. Next, we analyzed the effects of A_2A_R on the regulation of angiogenesis in human microvascular ECs (HMECs) *in vitro* and the role of c-Ski in this process by RNAi. Finally, the ERK1/2 antagonist PD98059 was used to confirm that A_2A_R regulates the expression of c-Ski through the ERK/CREB pathway *in vitro*.

## Materials and methods

### Animals

A_2A_R knockout (KO) mice (A_2A_R^-/-^) and their wild-type (WT) controls were bred on a C57BL/6 background. The A_2A_R^+/-^ breeding pairs were backcrossed to C57BL/6 mice for 10 generations, resulting in a congenic C57BL/6 genetic background. Heterozygous interbreedings (A_2A_R^+/-^ × A_2A_R^+/-^) were used so that the global A_2A_R homozygous KO mice (A_2A_R^-/-^) and their WT littermates were generated from the same breeding pairs 41. Mice with a 'floxed' adenosine A_2A_R gene (A_2A_^flox/flox^ mice) have been described previously^42^ and were provided by Dr. Chen. TeK-Cre transgenic mice (Tek, a strain that targets ECs)^43^, ^44^ were obtained from Shanghai Biomodel Organism Science & Technology Development Co., Ltd. (No. NMX-TG-192000, Shanghai, China). Global A_2A_R knockout mice, A_2A_^flox/flox^ mice and TeK-Cre transgenic mice were backcrossed for 10 to 12 generations to C57BL/6 mice. TeK-Cre transgenic mice were then crossed with A_2A_^flox/flox^ mice to generate TeK-A_2A_R KO mice and TeK-A_2A_R WT littermates, which were injected with 120 mg/kg tamoxifen (10540-29-1, Sigma Aldrich, St. Louis, MO, USA) in oil via intraperitoneal injection once every 48 hours 5 times in total, followed by a 14-day waiting period between the final injection and full-thickness wounding. The mice were used at 90 days of age. The animals were treated in accordance with the guidelines of the Animal Ethical and Welfare Committee of the Army Medical University and according to the protocol approved by the Administration of Affairs Concerning Experimental Animals Guidelines of The Army Medical University (No. AMUWEC20172073).

### Reagents

We purchased MCDB 131 medium (10372019), fetal bovine serum (FBS) (10099141C), TrypLE™ Express (12605028), penicillin‒streptomycin (15140122), and L-glutamine (25030081) from Fisher Scientific (Thermo Fisher, MA, USA). Hydrocortisone (M3451) was obtained from Abmole (Abmole, TX, USA), and recombinant mouse EGF protein (ab126695) was obtained from Abcam (Abcam, Cambridge, UK). The A_2A_R agonist 2-p-[2-carboxyethyl]phenethyl-amino-5'-N-ethylcarboxamido-adenosine (CGS21680) (1063) and the MEK inhibitor PD98059 (1213) were purchased from Tocris (Bio-Techne, MN, USA). Hydrocortisone, CGS21680, and PD98059 were prepared as stock solutions in dimethyl sulfoxide (DMSO), aliquoted, and stored at -80°C.

### Model and observation of wound healing

A mouse model of wound healing was generated as previously described with some modifications^26^. Briefly, to minimize discomfort and pain, mice were anesthetized with intraperitoneal injection of 50 mg/kg sodium pentobarbital, and two 1 cm diameter, full-thickness circular skin flaps were cut from the buttocks of the mice (at least six animals in each group). The wounds were covered with a piece of sterile gauze. The mice were then returned to their cages and maintained separately to avoid any further wound damage under a 12:12 h light/dark cycle, and supplied with unrestricted food and water.

Wound healing was observed as described previously^26^, ^45^. At 0, 3, 6, 9 and 15 d after wounding, wound closure was traced in each group using transparent film. And, percentage wound closure at each time point was derived by the formula: [1- (current wound area/original wound area)]×100%. The scar areas were calculated every 2 weeks using image processing software.

### Histological and quantitative image analysis

After perfusion, the mice were anesthetized with intraperitoneal injection of 50 mg/kg sodium pentobarbital and perfused with ice-cold normal saline and then with 4% paraformaldehyde solution. Next, the entire wound and its associated normal skin was excised and then was fixed overnight in 4% paraformaldehyde solution overnight for further fixation at 3, 6 and 9 d postwounding. After routine dehydration and paraffin embedding, the tissue sections (4 mm) were stained with hematoxylin and eosin (H&E) for morphological assessment. Re-epithelialization and granulation tissue formation were assessed as previously described^26^, ^45^.

Immunofluorescence was performed as previously described^46^, ^47^. Briefly, after dewaxing and antigen repair, primary antibodies were incubated with the samples overnight at 4°C in 0.01% Triton X-100 and 10% goat serum. The primary antibodies included goat anti-A_2A_R (1:200, Frontier Institute, AB_2571655), rabbit anti-CD31 (1:100, Servicebio, GB113151), rabbit anti-Ski (1:100, Santa Cruz, sc-9140), rabbit anti-CD11b (1:2000, Abcam, ab133357), rabbit anti-vimentin (1:400, Abway, CY5134), and rabbit anti-F4/80 (1:400, Cell Signaling, 70076). The tissue sections were washed with phosphate-buffered saline (PBS) and then treated with an Alexa Fluor 488-conjugated goat anti-rabbit antibody (1:500, Abcam, ab150077) and/or a Cy3-conjugated donkey anti-goat (1:500, Abcam, ab6949) antibody for 50 min at room temperature. The nuclei were stained with DAPI (Solarbio, C0060), and an autofluorescence quenching kit (Vector Laboratories, SP-8400) was used to remove unwanted fluorescence.

The results were captured by laser scanning confocal microscopy (TCS-SP8, Leica), and the quantitative analysis was performed independently by two observers who were blinded to the treatment conditions. The number of CD31-positive cells was counted and normalized to the total number of cells in each high-power field (HPF, 400×). The ratio of the integrated density (IntDen) to the total number of cells was determined with ImageJ software, and three fields were randomly chosen from each slice (three fields per section, three sections per mouse, five mice from each analyzed group).

### RNA isolation and RT‒PCR

At 0, 6, 9, and 15 d postwounding, total RNA was extracted from the full-thickness skin of the mouse wounds using a TRIzol Reagent RNA Extraction Kit (Invitrogen, 15596026) according to the manufacturer's instructions and then reverse transcribed to cDNA using a kit (Promega, A2791). A qPCR master mix kit (Promega, A600A) was used for quantitative PCR. The methods used were previously reported^48^. The primers for c-Ski were as follows: 5′-TCAACTCGGTGTGCGATG-3′ and 5′-CGTCCGTCTTGGTGATGAG-3′. The primers for GAPDH were as follows: 5′-AGGTTGTCTCCTGCGACTT-3′ and 5′-TGGTCCAGGGTTTCTTACTCC-3′. The samples were denatured by heating at 95°C for 30 s, followed by 40 cycles of 95°C for 30 s, 58°C for 30 s and 72°C for 30 s, and the relative abundance of the target gene was calculated via normalization to GAPDH.

### Western blotting

Western blotting was performed as previously described^46^. Briefly, at 0, 6, 9, and 15 d postwounding, the tissues were lysed in RIPA lysis buffer (Beyotime, P0013B) containing a protease and phosphatase inhibitor cocktail (Thermo Fisher, 78440). The total protein concentration was measured using a BCA kit (Aidlab, PP0102). The samples were resolved on 10% SDS‒PAGE and then transferred to polyvinylidene fluoride (PVDF) membranes (Millipore, IPVH00010). After the membranes were blocked with 5% bovine serum albumin (Solarbio, A8020) in Tris-buffered saline (pH 7.6) containing 0.1% Tween-20 (TBS-T) at room temperature for 1 hour, they were probed with the following primary antibodies overnight at 4°C: anti-Ski (1:100, Santa Cruz, sc-9140), anti-A2A adenosine receptor (1:1000, Abcam, ab3461), and anti-GAPDH (1:5000, Abcam, ab9485). After 3 washes with TBS-T incubation for 1 h at room temperature with HRP-conjugated goat anti-mouse IgG or goat anti-rabbit IgG secondary antibodies (1:8000, Abways, AB0035), the membranes were visualized using Clarity Western ECL Substrate (Beyotime, P0018). The band intensity was quantified by ImageJ software, and the relative quantity of the target protein was normalized to that of GAPDH or the nonphosphorylated protein for three independent experiments.

### Cell culture and treatment

HMEC-1 cells were provided by Dr. Lan^49^ and were characterized by Feiouer Biotechnology Co., Ltd. (Chengdu, China) using short tandem repeat (STR) markers (Fig. S9). The cells were maintained at 37°C at 5% CO_2_ in MCDB 131 medium supplemented with 10% FBS, 10 ng/mL recombinant mouse EGF protein, 1 μg/mL hydrocortisone, 1% penicillin‒streptomycin, and L-glutamine.

To determine the possible signaling mechanism, the cells were treated with 20 nM, 100 nM, or 500 nM of the A_2A_R agonist CGS21680, which was dissolved in DMSO, for 24 h or were pretreated with 100 µM of the MEK inhibitor PD98059 for 60 min before 100 nM CGS21680 treatment according to the protocol previously described. DMSO was used as the vehicle control.

### Lentivirus infection

Short hairpin RNA (shRNA)-knockdown lentiviral particles for mouse c-Ski (sh c-Ski) and short hairpin negative control (sh Nc) lentiviruses were purchased from Shanghai Jikai Company (Shanghai, China). For c-Ski knockdown experiments, HMEC-1 cells were transfected with 5 nM, 10 nM, or 20 nM sh c-Ski for 24 h following the manufacturer's instructions before 100 nM CGS21680 treatment for 24 h.

### Cell proliferation and scratch assays

A Cell Counting Kit (CCK)-8 assay (Beyotime, C0038) was used to assess cell proliferation^47^. Briefly, HMEC-1 cells were trypsinized using TrypLE™ Express and centrifuged at 1000g for 5 min at room temperature and then were seeded in 96-well plates with 100 μL of medium at a density of 8,000 cells/well. After various treatments, 10 µL of CCK-8 solution was added to each well and incubated for 2 h at 37°C and 5% CO_2_. The optical density (OD) values were measured at 450 nm using a microplate reader (Gene, ELX800ux). Wells without cells served as blank controls. Each experiment was performed in triplicate.

The scratch assay was performed as previously described^49^. Briefly, HMEC-1 cells were seeded in 6-well plates. The monolayer was scratched with a 200 μL pipette tip and rinsed with PBS after various treatments. Images were taken under an inverted phase contrast microscope (Zeiss, Primo Vert) with CCD cameras at 0, 24, 48, and 72 h after scratching. The experiment was repeated independently three times.

### Tube formation

Matrigel (Corning, 356,234) was added to a precooled Millicell EZ SLIDE 8-well glass slide (200 μL/well) and polymerized for 40 min at 37°C. HMEC-1 cells were grown to 80% confluence and trypsinized using TrypLE™ Express. 1 × 10^4^ cells in each treatment group were seeded on the Matrigel, incubated for 4 h in the corresponding medium at 37°C during tubule formation and then and washed with Hank's balanced salt solution (HBSS). For CGS21680 treatment, the cells were treated with calcein AM fluorescent dye (1:300, Corning, 354,216) for 4h. For CGS21680 and c-Ski RNAi treatment, the cells were treated with DiIC12(3) fluorescent dye (1:1000, Corning, 354,218). The results were captured by laser scanning confocal microscopy (TCS-SP8, Leica), and the number of junctions of the tubular-like structures was determined via Angiotool software 50. Three independent experiments were performed.

### *In vitro* immunofluorescence and western blotting

*In vitro* immunofluorescence and western blot assays were performed according to previously described methods. Briefly, for immunofluorescence analyses, HMEC-1 cells were fixed with 4% paraformaldehyde for 10 minutes and then incubated with the primary antibodies goat anti-A_2A_R (1:200, Frontier Institute, AB_2571655), rabbit anti-Ski (1:100, Santa Cruz, sc-9140), anti-p-PKA (1:500, Abcam, ab32390), anti-p-ERK1/2 (1:200, Cell Signaling, 9101), and anti-p-CREB (1:500, Cell Signaling, 9198) at 4°C overnight. The cells were then rinsed with phosphate-buffered saline (PBS) and treated with an Alexa Fluor 488-conjugated goat anti-rabbit antibody or a Cy3-conjugated donkey anti-goat antibody for 1 h at 37°C. The nuclei were stained with DAPI. The results were captured by laser scanning confocal microscopy (TCS-SP8, Leica), and the quantitative analysis was performed independently by two observers who were blinded to the treatment conditions. The ratio of the integrated density (IntDen) to the total number of cells was determined via ImageJ software, and three fields were randomly chosen from each well (five wells per group and 3 independent experiments).

For western blot assays, HMEC-1 cells were seeded into 25 cm^2^ vented cap flasks. After various treatments, the whole-cell lysates were collected and then separated on a 10% SDS‒polyacrylamide gel. After blocking with 5% BSA in Tris-buffered saline (pH 7.6) containing 0.1% Tween 20 (TBS-T) at room temperature for 1 hour, the PVDF membranes were probed with the following primary antibodies overnight at 4°C: anti-Ski (1:100, Santa Cruz, sc-9140), anti-A2A adenosine receptor (1:1000, Abcam, ab3461), anti-PKA (1:1000, Cell Signaling, 4782), anti-p-PKA (1:1000, Abcam, ab32390), anti-CREB (1:300, Cell Signaling, 9197), anti-p-CREB (1:1000, Cell Signaling, 9198), anti-ERK1/2 (1:1000, Cell Signaling, 9102), anti-p-ERK1/2 (1:1000, Cell Signaling, 9101), and anti-GAPDH (1:5000, Abcam, ab9485). After incubation with HRP-conjugated goat anti-mouse IgG or goat anti-rabbit IgG secondary antibodies (1:8000, Abways, AB0035), the membranes were washed with TBS-T 3 times and visualized using Clarity Western ECL Substrate (Beyotime, P0018). The band intensity was quantified by ImageJ software, and the relative quantity of the target protein was normalized to that of GAPDH or the nonphosphorylated protein for three independent experiments.

### Statistical analysis

All procedures and analyses were performed by an experienced researcher who was blinded to all groups. All of the results are expressed as the means ± standard errors of the means. Statistical analyses were performed using GraphPad Prism 6 software (San Diego, CA, USA). Two-group comparisons were performed using Student's t test. One-way and two-way analysis of variance (ANOVA) was used for data with one variable and multiple conditions, followed by Tukey's multiple comparisons test. A value of *P* < 0.05 was considered statistically significant.

## Results

### A_2A_R expression in full-thickness excisional wounds

**Figure 1:**
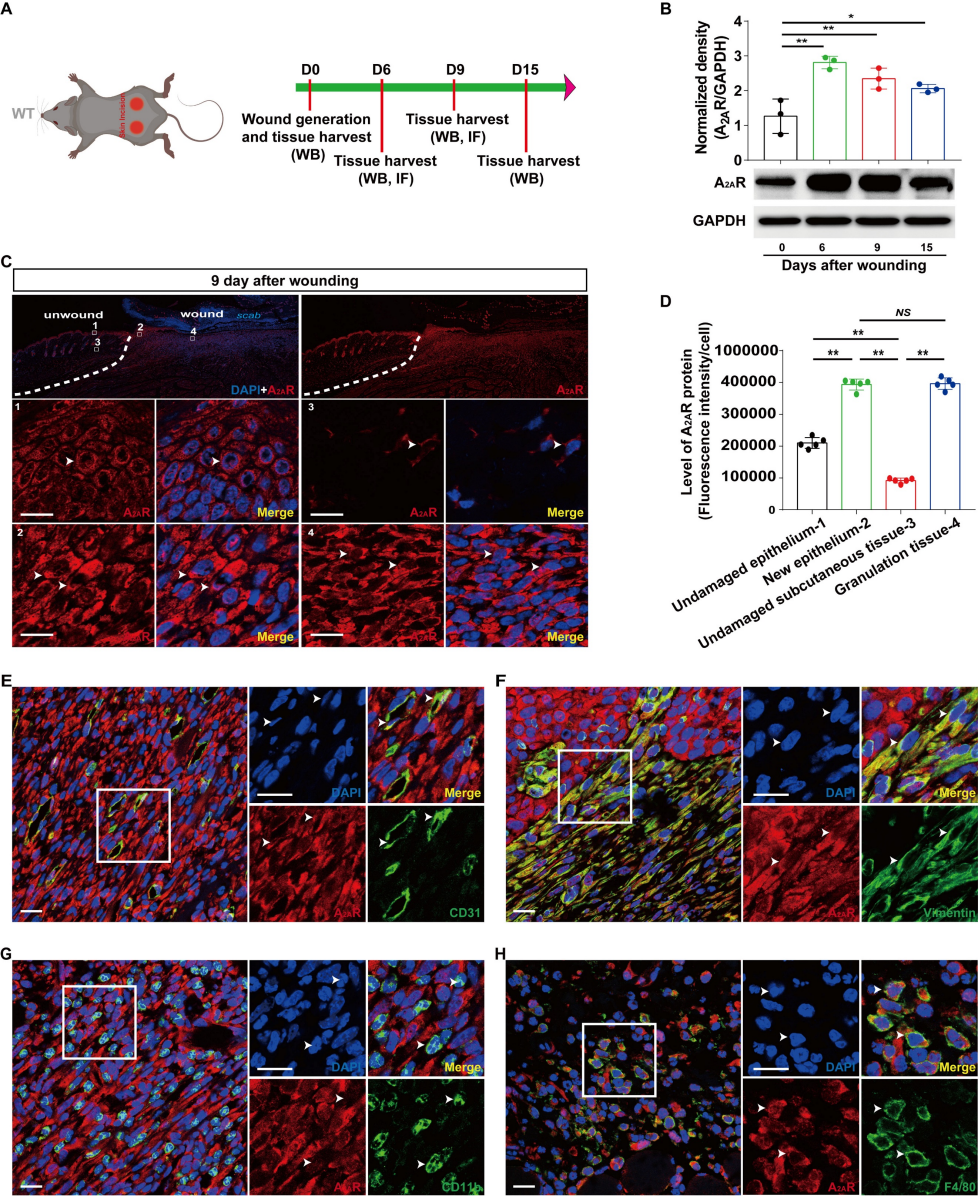
** Localization and expression of A_2A_R in full-thickness excisional wounds of WT mice.** (A) Experimental procedure. (B) A_2A_R levels were detected by western blotting at 0, 6, 9 and 15 days postwounding. **p < 0.01, *p <0.05 (n=3); NS, not significant. Immunohistochemistry for A_2A_R (red, white arrow) in the wound and surrounding area at 9 days postwounding (C) and its quantitative analysis (D) in WT mice. **p < 0.01 (n=5); NS, not significant. The lower panel shows higher magnification sections of the white squares in the upper panel. Scale bar, 50 µm. Immunohistochemistry for CD31 (E), vimentin (F), CD11b (G), F4/80 (H) (green) and A_2A_R (red) in wound granulation tissue at 9 days postwounding in WT mice. The right panel shows higher magnification sections of the white squares on the left, and the white arrow indicates that the green fluorescent-labeled cells colocalized with the red fluorescent-labeled cells. Short scale bar, 50 µm; long scale bar, 50 µm.

To study the role of A_2A_R in wound healing, we assessed the expression profile of A_2A_R in mouse dermal excisional wounds (Fig. 1A). Western blot analyses revealed that A_2A_R expression began to increase after the injury, peaked at 6 days, and was maintained at a high level during the late stages of wound healing (Fig. 1B). Next, immunofluorescence staining revealed that A_2A_R was widely distributed in the undamaged epidermis and subcutaneous tissue around the wound, resulting in a hole-like morphology (Fig. 1C), similar to our previous results^39^, ^42^. Moreover, A_2A_R expression increased in the epidermis and granulation tissue in the wound at 9 days postwounding and was especially prominent in granulation tissue (Fig. 1C-D). To characterize the expression of A_2A_R in granulation tissue further, we performed double immunofluorescence staining and revealed that A_2A_R was widely expressed in repair cells; was especially prominent in CD31^+^ ECs, vimentin^+^fibroblasts and F4/80^+^ macrophages; and was expressed at low levels in CD11b^+^ inflammatory cells (Fig. 3E-G). Thus, changes in the expression of A_2A_R during wound healing and its localization in repair cells suggest that this molecule is involved in the regulation of healing.

### Both A_2A_R KO and EC-A_2A_R KO delay full-thickness excisional wound healing and inhibit granulation tissue formation

To directly confirm the role of A_2A_R in angiogenic regulation during wound healing through ECs, we used A_2A_R-KO mice and A_2A_R flox^+/+^ Tek-Cre^+^ mice (EC-A_2A_R KO and Tek-Cre^+^ mice were bred with A_2A_R^flox/flox^ mice for a generation). Western blot analysis revealed that A_2A_R protein was not detected in the A_2A_R-KO mice (Fig. S1A), and its level was slightly lower in the EC-A_2A_R-KO mice compared with the A_2A_R flox^+/+^ Tek-Cre^-^ control (EC-A_2A_R control) mice (Fig. S1B). Furthermore, immunofluorescence staining revealed that the A_2A_R protein level in CD31-positive cells was not significantly different between the wild-type (WT) and EC-A_2A_R control mice in undamaged subcutaneous tissue or in the granulation tissue of the wound at 9 days post-wounding (Fig. S1C and S2). In contrast, A_2A_R protein was not detected in any cells from the A_2A_R-KO mice and was found only in CD31-positive cells from the EC-A_2A_R-KO mice (Fig. S1A-C and S2). Collectively, these results suggest that the A_2A_R-KO and EC-A_2A_R-KO mice were successfully established.

**Figure 2:**
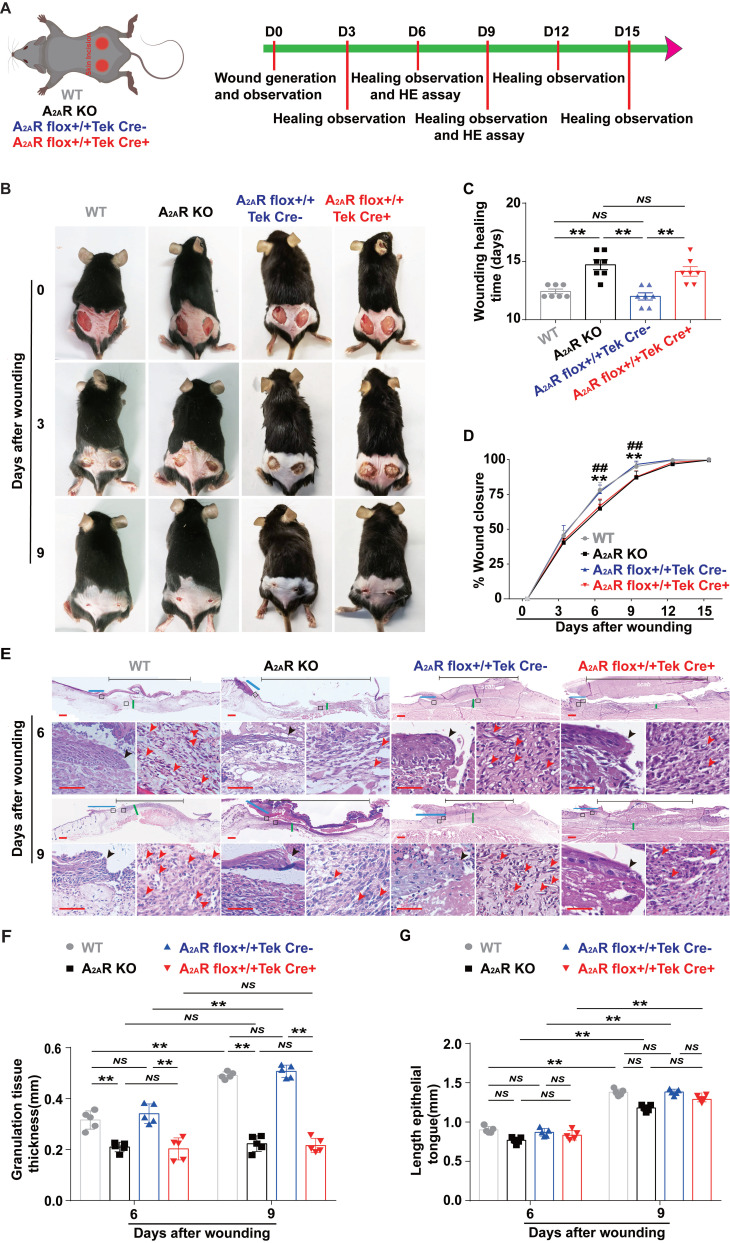
** Wound healing and histopathological characteristics of A_2A_R KO and EC-A_2A_R KO mice after full-thickness wounding.** (A) Experimental procedure. (B) Photographs of representative wounds of mice at 0, 3 and 9 days postwounding. (C) Wound healing time for each experimental and control group (n = 7 for each group), **p < 0.01. (D) Time course of wound closure for each experimental and control group (n = 9 for each group). **p <0.01, compared with the appropriate controls; ^##^*p* <0.01, compared with the previous adjacent time point. (E) Histopathological observation of wound healing at 6 and 9 days postwounding. Short scale bar, 200 µm; long scale bar, 50 µm. (F) Quantitative analysis of granulation tissue thickness at 6 and 9 days postwounding. **p < 0.01 (n = 5); NS, not significant. (G) Quantitative analysis of the crawling distance at 6 and 9 days postwounding; **p < 0.01 (n = 5); NS, not significant.

Next, we assessed the effects of A_2A_R-KO and EC-A_2A_R KO on wound healing using full-thickness excisional wounds as described in a previous experiment^26^, ^45^ (Fig. 2A). Comparison of the wound size revealed a clear delay in wound healing in the A_2A_R-KO and EC-A_2A_R-KO mice compared with the WT and EC-A_2A_R control mice, particularly at 9 days postwounding (Fig. 2B). Moreover, there were no significant differences in the rate of wound closure or healing time between the WT and EC-A_2A_R control mice, while both the A_2A_R-KO and EC-A_2A_R-KO mice presented a decreased rate of wound closure and delayed healing time (Fig. 2C-D). Furthermore, there was no significant difference in the rate of wound closure or healing time between the A_2A_R-KO and EC-A_2A_R-KO mice (Fig. 2C-D). Collectively, these results demonstrate that the delayed wound healing induced by A_2A_R KO is due mainly to the endothelial A_2A_R effect and suggest that A_2A_R-mediated angiogenesis plays a critical role in wound healing.

Additionally, H&E-stained sections revealed that granulation tissue grew significantly slower in both the A_2A_R-KO and EC-A_2A_R-KO mice than in the WT and EC-A_2A_R control mice at 6 and 9 days post wounding (Fig. 2E-F). Moreover, there was no significant difference in granulation tissue formation between the A_2A_R-KO and EC-A_2A_R-KO mice or the WT and EC-A_2A_R control mice (Fig. 2E-F). Conversely, there was no significant difference in re-epithelialization at 6 and 9 days postwounding in any group, including the WT, A_2A_R-KO, EC-A_2A_R control and EC-A_2A_R-KO mice (Fig. 2E and G). However, there was an obvious decrease in tube-like structures in both A_2A_R-KO and EC-A_2A_R-KO mice (Fig. 2E). Taken together, these results suggest that A_2A_R plays an important role in granulation tissue formation, but not epithelialization, and it is possible that A_2A_R affects granulation tissue formation mainly by regulating angiogenesis.

### Both A_2A_R KO and EC-A_2A_R KO decrease angiogenesis in the granulation tissue of full-thickness excisional wounds and the expression of c-Ski

**Figure 3:**
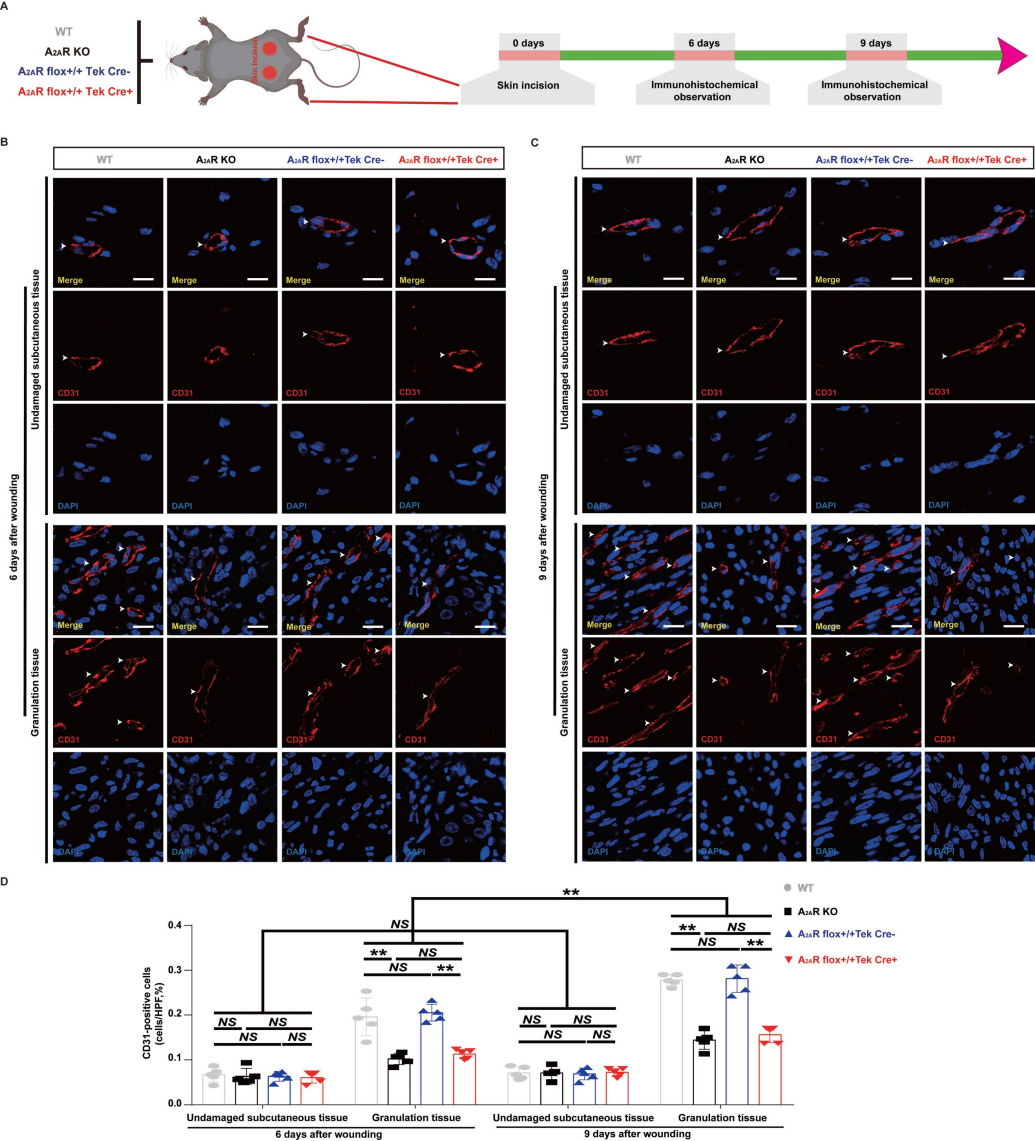
** Changes in angiogenesis in A_2A_R KO and EC-A_2A_R KO mice after full-thickness wounding.** (A) Experimental procedure. (B) Immunohistochemistry for CD31 in A_2A_R KO and EC-A_2A_R KO mice at 6 and 9 days postwounding. Scale bar, 50 µm. (C) Quantitative analysis of CD31-positive cells in each group. **p < 0.01 (n = 3); NS, not significant.

Previous studies have indicated that adenosine receptor-mediated stimulation of angiogenesis promotes wound closure in mice treated with the adenosine receptor agonist CGS21680 and A_2A_R-KO mice^15^, ^51^. We first used EC-A_2A_R KO mice to examine the vascularity of wounds in total KO, EC-specific A_2A_R KO and control mice (Fig.3A and S4A). We observed a nearly twofold decrease in CD31-positive cells in both the A_2A_R-KO and EC-A_2A_R-KO mice compared with the WT and EC-A_2A_R control mice at 6 and 9 days postwounding (Fig. 3B-D, S2, S3A-B, S4B), but the differences between the A_2A_R-KO and EC-A_2A_R-KO mice or the WT and EC-A_2A_R control mice were no longer significant (Fig. 3B-D, S2, S4B). Furthermore, the number of CD31-positive cells in the A_2A_R-KO or EC-A_2A_R-KO mice decreased to a certain extent between 6 and 9 days postwounding but did not significantly differ (Fig. 3B-D). Additionally, the number of CD31-positive cells in the undamaged subcutaneous tissue of these four groups was not significantly different (Fig. 3B-D). These results suggest that A_2A_R affects granulation tissue formation mainly by regulating angiogenesis.

**Figure 4:**
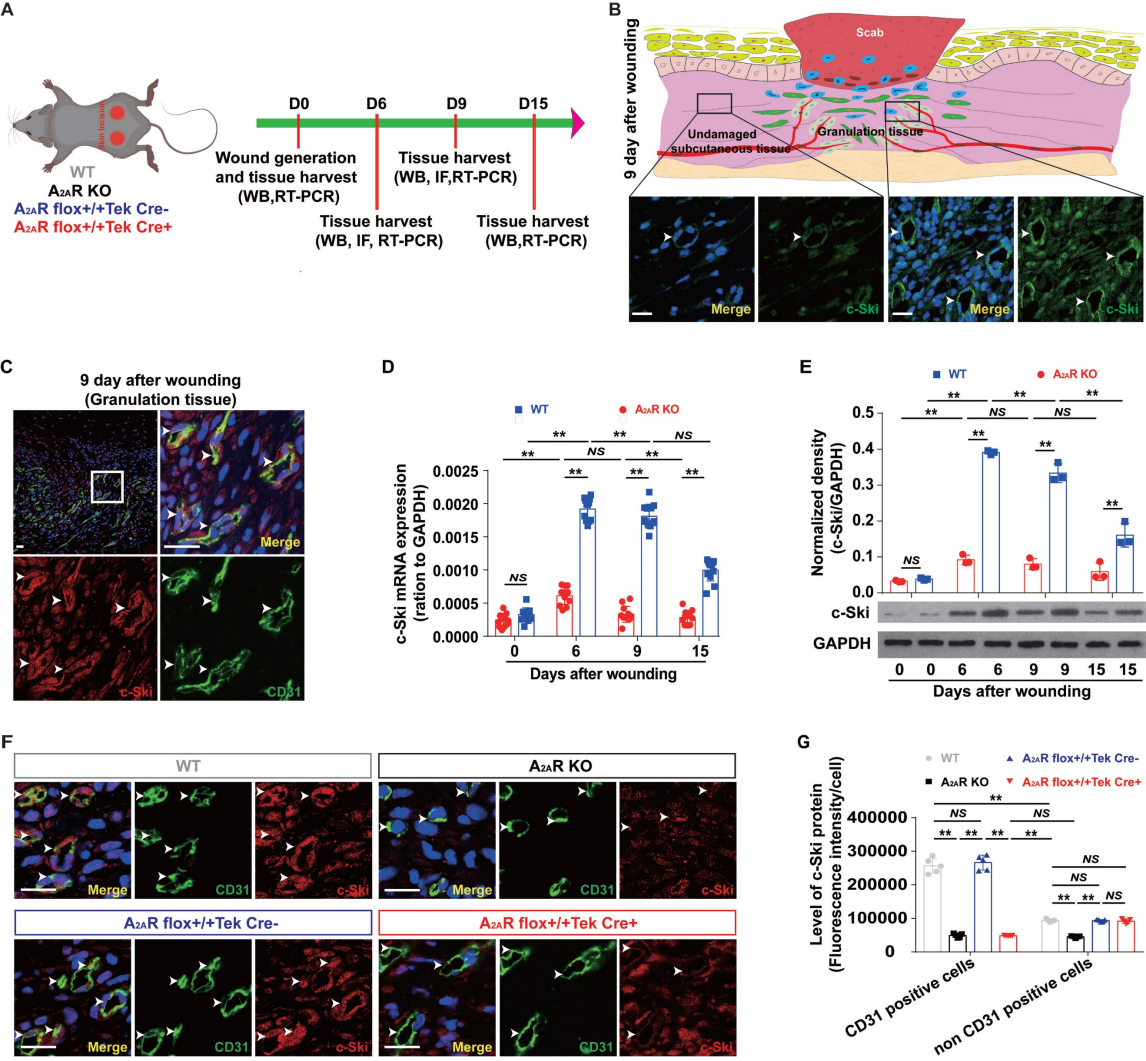
** The expression of c-Ski in A_2A_R KO and EC-A_2A_R KO mice after full-thickness wounding.** (A) Experimental procedure. (B) Immunohistochemistry for c-Ski (green, white arrow) in the granulation tissue of the wound and subcutaneous tissue of the surrounding wound at 9 days postwounding. The lower panel shows higher magnification sections of the black squares in the upper panel. Scale bar, 50 µm. (C) Double-label immunofluorescence for c-Ski (red) and CD31 (green) in the granulation tissue of the wound at 9 days postwounding revealed the colocalization of c-Ski and CD31 (white arrow). The surrounding area is a higher magnification section of the white square. Short scale bar, 50 µm; long scale bar, 50 µm. Expression of c-Ski detected by (D) real-time PCR (n = 9) and (E) western blot analysis (n = 3) at 0, 6, 9 and 15 days postwounding. **p < 0.01; NS, not significant. (F) Double-label immunofluorescence for c-Ski (red) and CD31 (green) in the granulation tissue of the wound at 9 days postwounding in each group. The white arrow indicates the colocalization of c-Ski and CD31. Scale bar, 50 µm. (G) Quantitative analysis of (F). **p < 0.01 (n=5); NS, not significant.

To determine whether the deletion of A_2A_R affects angiogenesis during wound healing through c-Ski, we analyzed the effects of A_2A_R on the expression of c-Ski in A_2A_R-KO and EC-A_2A_R-KO mice (Fig.4A and S5A). Consistent with our previous results^39^, ^42^, the level of c-Ski in the wound area was greater than that in the surrounding area (Fig. 4B and S5B), and c-Ski expression significantly increased after injury and was maintained at a high level during the late stages of wound healing (Fig. 4D-E). Furthermore, immunofluorescence revealed that the c-Ski level in CD31-positive cells was greater than that in non-CD31-positive cells at 9 days post wounding (Fig. 4C and S5B). Moreover, c-Ski was colocalized with A_2A_R in both undamaged subcutaneous tissue and the granulation tissue of the wound (Fig. S5C). In contrast to WT mice, the expression and level of c-Ski were significantly reduced in A_2A_R-KO mice after wounding (Fig. 4D-E). Immunofluorescence staining further confirmed that the c-Ski protein level in CD31-positive cells was not significantly different between the WT and EC-A_2A_R control mice (Fig. 4F-G), whereas c-Ski protein expression was significantly decreased in all cells of the A_2A_R-KO mice and was found only in CD3-positive cells of the EC-A_2A_R-KO mice (Fig. 4F-G and S6). Collectively, these results suggest that A_2A_R regulates angiogenesis through c-Ski.

### A_2A_R activation induces a proangiogenic effect, as shown by the application of an A_2A_R agonist *in vitro*

**Figure 5:**
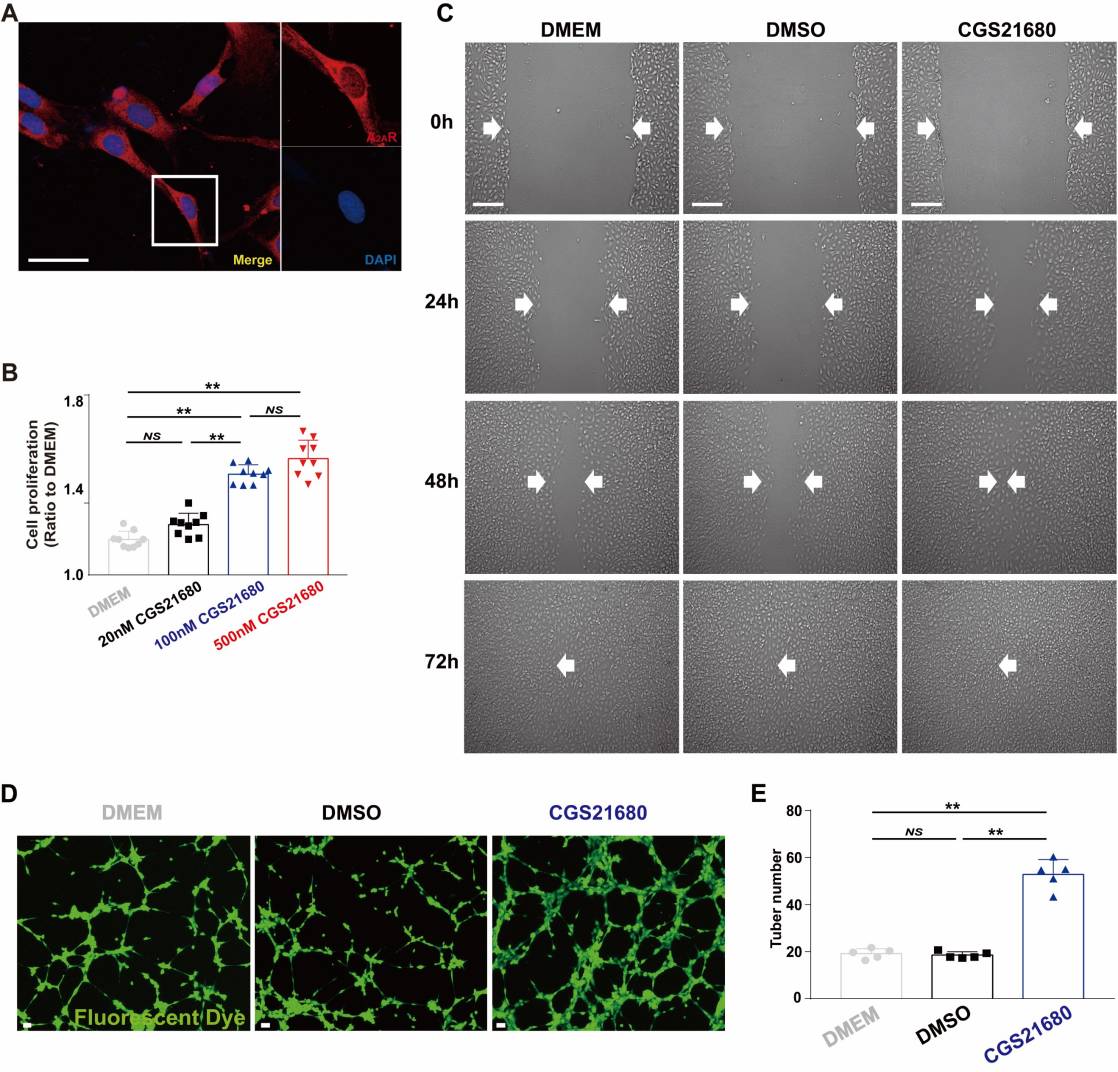
** Changes in the proangiogenic effect in HMECs by the application of an A_2A_R agonist *in vitro*.** (A) Immunohistochemistry of A_2A_R (red) in HMECs. Scale bar, 50 µm. (B) Cell proliferation was measured by the CCK-8 assay 24 h after treatment with different concentrations of CGS21680. **p < 0.01 (n=9); NS, not significant. (C) Representative images of cell migration evaluated by scratch wound healing assays at 0, 24, 48 and 72 h after CGS21680 treatment. Scale bar, 50 µm. (D) Representative images of tube formation 24 h after treatment with CGS21680. Scale bar, 50 µm. (E) Quantitative analysis of (D). **p < 0.01 (n=5); NS, not significant.

After confirming the essential role of angiogenesis in A_2A_ R deficiency-mediated impairment of wound healing involving c-Ski, we further verified the proangiogenic effect of A_2A_ R *in vitro*, laying the foundation for future exploration of the role of c-Ski in this effect. Previous studies have indicated that adenosine, which acts through A_2A_R, promotes angiogenesis in pulmonary ECs^52^-^54^, vascular ECs^55^, ^56^, retinal ECs^57^, umbilical vein ECs^58^, lymphatic ECs^59^, and human EA.hy926 ECs^60^. However, the activation of A_2A_R does not affect angiogenesis in bone fracture medium^55^ or human lung microvascular ECs^61^. We used HMECs, a critical component of granulation tissue involved in wound healing, to verify the proangiogenic effect induced by A_2A_R activation via cell proliferation, migration and tube formation assays, which are often used to evaluate angiogenesis *in vitro*^53^, ^62^, ^63^. Immunofluorescence staining revealed that A_2A_R was expressed in HMECs and exhibited a hole-like morphology (Fig. 5A). Moreover, application of the A_2A_R agonist CGS21680 promoted cell proliferation in a dose-dependent manner (Fig. 5B). In the scratch assay, cell migration was obviously greater in the CGS21680 group than in the DMEM and DMSO control groups (Fig. 5C). Moreover, CGS21680 treatment resulted in a significant increase in the tube formation of HMECs compared with that of the DMEM and DMSO control groups in angiogenesis assays *in vitro* (Fig. 5D-F). Consistent with other studies^53^, ^54^, ^56^, ^64^, the activation of A_2A_R by CGS21680 significantly promoted the proliferation, migration and angiogenesis of HMECs.

### A_2A_R regulates angiogenesis by regulating c-Ski, as shown by RNAi of c-Ski expression in HMECs

**Figure 6:**
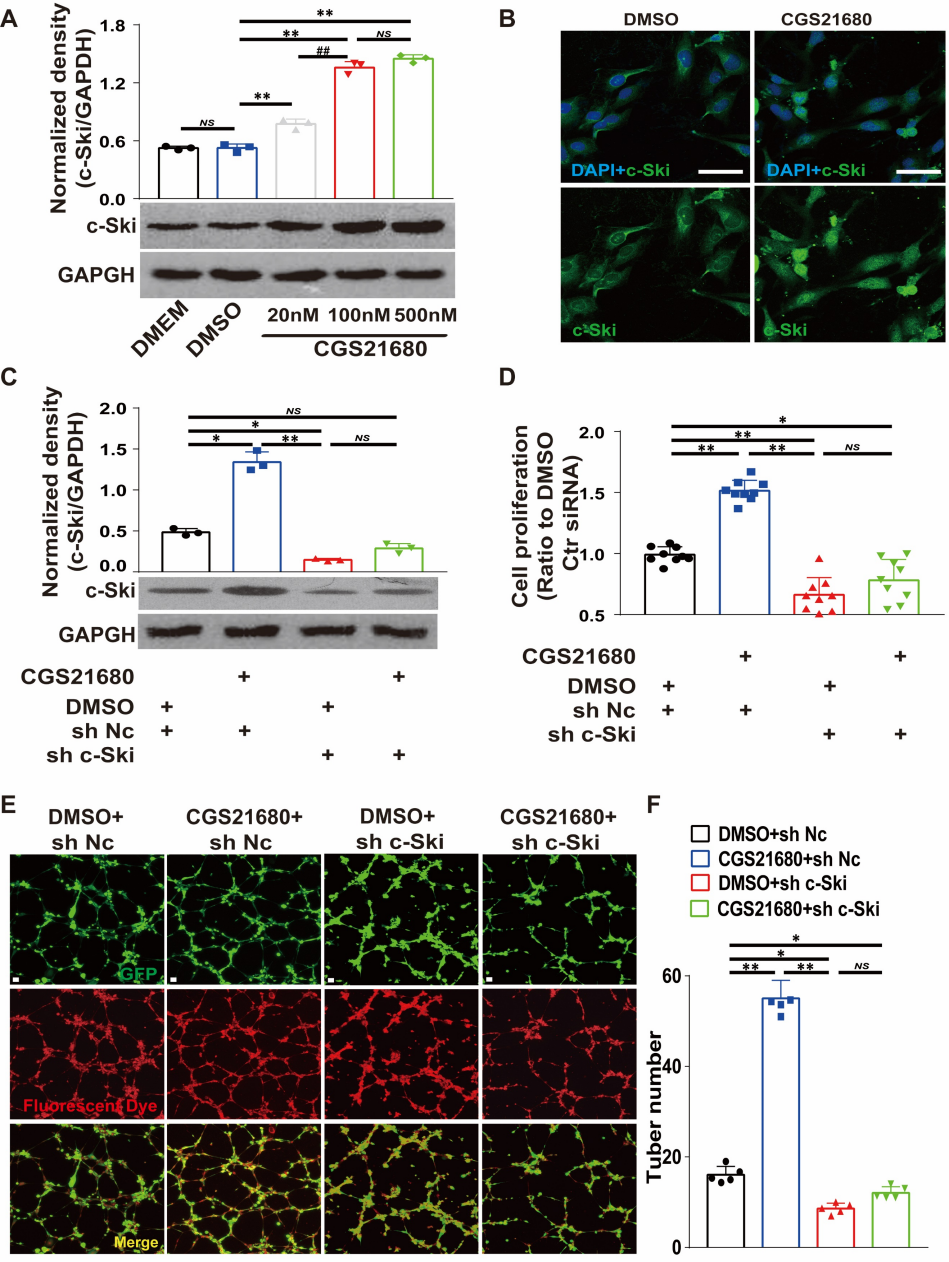
** Effects of c-Ski on the proangiogenic effect of A_2A_R activation in HMECs.** (A) Levels of c-Ski detected by western blots 24 h after treatment with different concentrations of CGS21680. ** p < 0.01, ^##^*p* <0.01, compared with the previous adjacent group (n = 3); NS, not significant. (B) Immunohistochemistry for c-Ski (green) in HMECs 24 h after CGS21680 treatment. Scale bar, 50 µm. (C) Changes in the CGS21680-induced increase in the intracellular protein level of c-Ski in HMECs after 24 h of c-Ski RNAi. ** p < 0.01, *p <0.05 (n = 3); NS, not significant. (D) Changes in CGS21680-induced cell proliferation for 24 h in HMECs after c-Ski RNAi. ** p < 0.01 (n = 9); NS, not significant. (E) Representative images of CGS21680-induced tube formation in HMECs after c-Ski RNAi treatment for 24 h. Scale bar, 50 µm. (F) Quantitative analysis of (E). **p < 0.01, *p <0.05 (n=5); NS, not significant.

To determine whether c-Ski is required for A_2A_R-mediated angiogenesis, we used CGS21680 to activate A_2A_R and RNAi to inhibit the expression of c-Ski in HMECs. Transfection with sh c-Ski successfully reduced the protein level of c-Ski in a dose-dependent manner (Fig. S8A-B), and CGS21680 treatment increased the c-Ski protein level in a dose-dependent manner (Fig. 6A). Immunofluorescence staining revealed that the c-Ski protein level increased after CGS21680 treatment, and c-Ski clearly aggregated in the nucleus (Figs. 6B and S7A). Interestingly, the c-Ski protein level was greater in dividing cells (Fig. S7B), suggesting an important role of c-Ski in cell proliferation. RNAi not only significantly reduced the level of the c-Ski protein compared with that of the control but also significantly reduced the increase in the c-Ski protein induced by CGS21680 treatment (Fig. 6C). Similarly, c-Ski depletion reduced the proliferation and tube formation of HMECs and abolished the effects of CGS21680 treatment on promoting cell proliferation and tube formation (Fig. 6D-F). Overall, these cell proliferation and tube formation assays strongly indicate that c-Ski plays an essential role in A_2A_R-mediated angiogenesis in HMECs.

### A_2A_R regulates the expression of the c-Ski protein through the ERK/CREB pathway in HMECs

**Figure 7:**
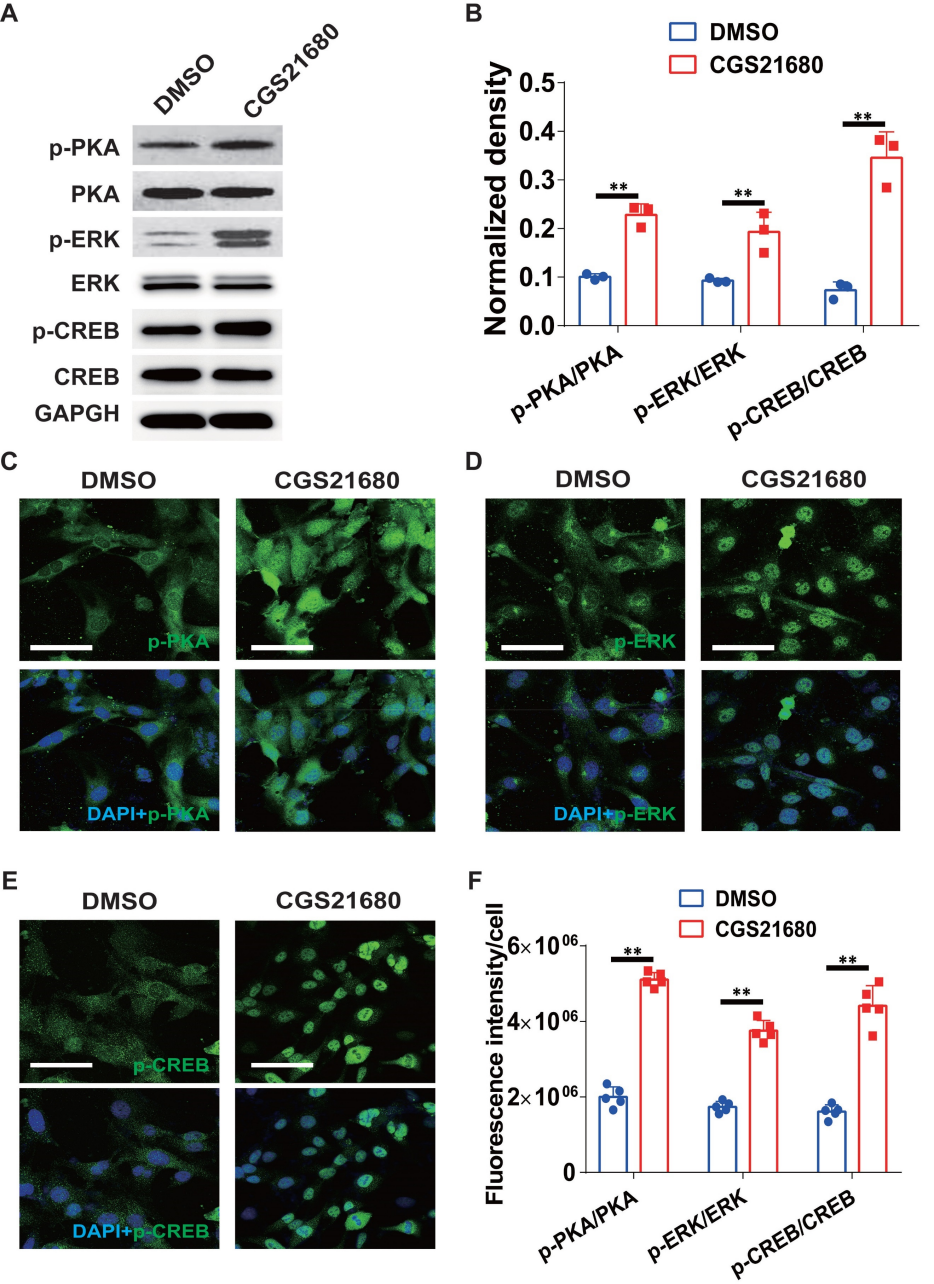
** Changes in the ERK/CREB signaling pathways after A_2A_R activation in HMECs.** (A) Representative immunoblot images of the levels of PKA, p-PKA, ERK, p-ERK, CREB and p-CREB in HMECs 24 h after treatment with CGS21680. (B) Quantitative analysis of (A). **p < 0.01 (n=3); Immunohistochemistry for p-PKA (C), p-ERK (D) and p-CREB (E) in HMECs 24 h after treatment with CGS21680. Scale bar, 50 µm. (F) Quantitative analysis of (C-E). **p < 0.01, *p <0.05 (n=5).

To characterize the underlying mechanisms by which A_2A_R regulates the expression of the c-Ski protein, we surveyed the ERK/CREB signaling pathway. As shown in Fig. 7A-B, CGS21680 treatment significantly increased the PKA, ERK and CREB phosphorylation levels in HMECs. Similar results were obtained by immunofluorescence staining at 24 h after CGS21680 treatment, indicating that activation of A_2A_R by CGS21680 can enhance the PKA/ERK/CREB signaling pathways in HMECs (Fig. 7C-F).

**Figure 8:**
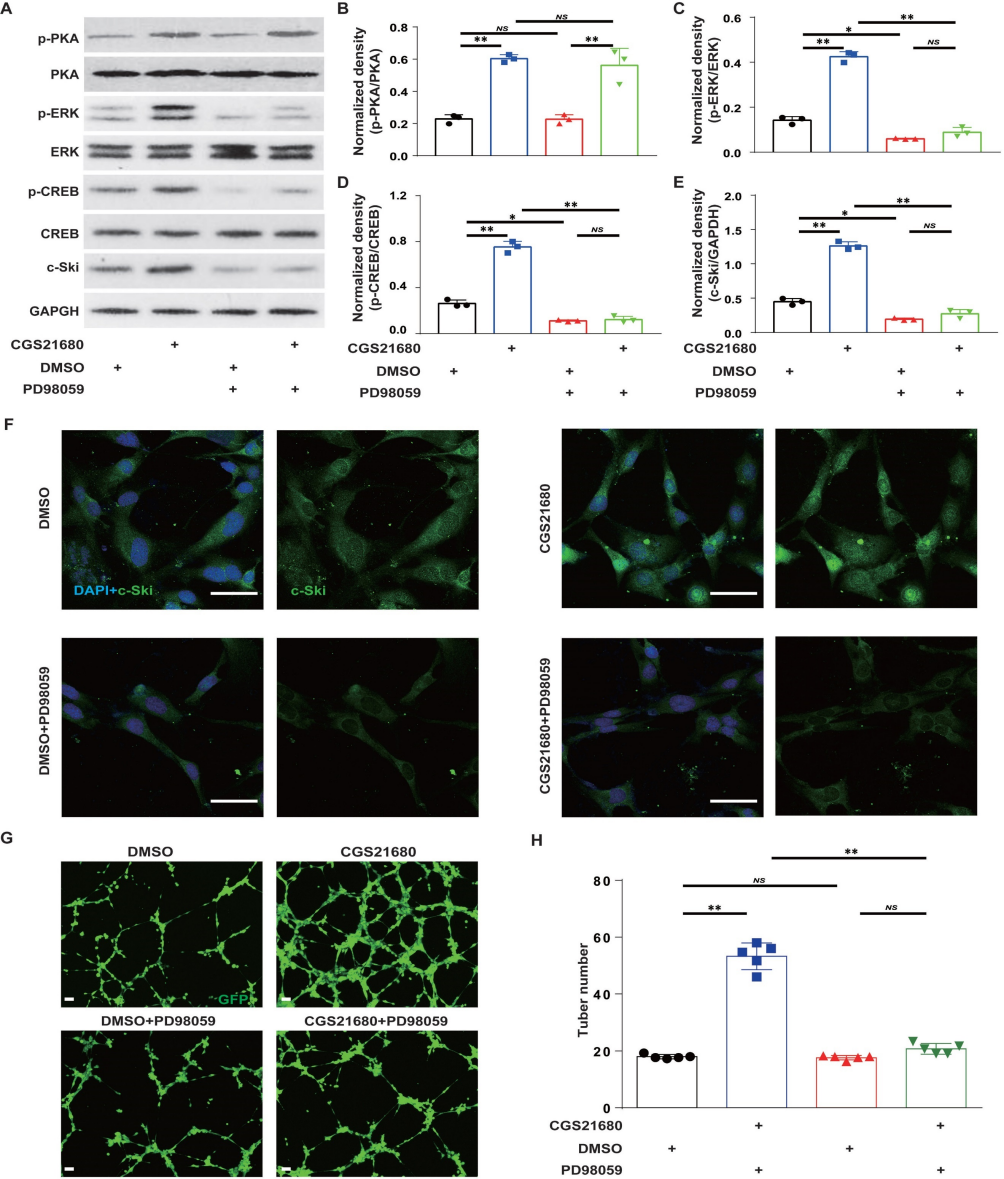
** Effects of the ERK/CREB signaling pathways on A_2A_R-induced expression of c-Ski in HMECs.** Representative immunoblot images of the CGS21680-induced changes in the intracellular protein levels of PKA, p-PKA, ERK, p-ERK, CREB and p-CREB in HMECs after ERK/CREB inhibition. (B-E) Quantitative analysis of (A). **p < 0.01 (n=3); NS, not significant. (F) Immunohistochemistry for c-Ski (green) induced by treatment with CGS21680 in HMECs after ERK/CREB inhibition. Scale bar, 50 µm. (G) Representative images of tube formation 24 h after treatment with CGS21680. Scale bar, 50 µm. (H) Quantitative analysis of (G). **p < 0.01 (n=5); NS, not significant.

To further verify that A_2A_R regulates c-Ski protein expression through the ERK/CREB pathway in HMECs, we used CGS21680 to activate A_2A_R and the MEK1/2 inhibitor PD98059^40^, ^65^ to inhibit ERK activity in HMECs. Compared with the lack of effect on p-PKA (Fig. 8A-B), PD98059 alone significantly reduced p-ERK, p-CREB and c-Ski levels and abrogated the CGS21680 treatment-induced increases in p-ERK, p-CREB and c-Ski levels (Fig. 8A and C-E). Similarly, immunofluorescence staining revealed that PD98059 not only decreased the fluorescence intensity of c-Ski but also abrogated the CGS21680 treatment-induced increase in the fluorescence intensity of c-Ski (Fig. 8F). Moreover, compared with DMSO, PD98059 not only decreased the tube formation of HMECs but also abolished the CGS21680 treatment-induced increase in the tube formation of HMECs in angiogenesis assays *in vitro* (Fig.8G-H). Taken together, these results suggest that A_2A_R regulates c-Ski protein expression in HMECs through the ERK/CREB pathway.

## Discussion

Some studies have shown that A_2A_R is expressed on most cell types involved in wound healing, including macrophages, fibroblasts and microvascular ECs, *in vitro*^8^, ^18^, ^61^, ^66^. We first explored the types of expression in these repair-associated cells *in vivo*. According to the histochemical results of full-thickness excisional wounds, A_2A_ R was expressed on most repair cells, including epidermal cells, ECs, macrophages and fibroblasts (Fig. 1), suggesting that it regulates wound healing. Interestingly, the increase was greater in the granulation tissue of the wound than in the epidermis relative to the undamaged side, suggesting that regulating granulation tissue formation is more important than re-epithelialization in A_2A_R regulation of wound healing, which is consistent with the findings of some studies^15^, ^54^. Importantly, the peak expression level of A_2A_R during wound healing occurred at 6 days postwounding (Fig. 1), which is also the fast period of wound healing (Fig. 2)^67^-^69^. In contrast, A_2A_R protein levels are lower at 7 days postwounding than at 3 days during the healing of refractory wounds in diabetic mice^70^. Taken together, these results suggest that A_2A_R may have a wound-promoting effect. A_2A_R agonists can promote skin wound healing and radiation-impaired wound healing^8^-^13^, whereas A_2A_R gene knockout delays the course of skin wound healing^10^, ^15^, ^71^, which also indicates the promotion of healing by A_2A_R.

Similar to the findings of a previous experiment^15^, ^54^, our study revealed that A_2A_R KO delayed wound healing compared with WT mice. Importantly, there was no significant difference in the rate of wound closure or healing time between the A_2A_R-KO and EC-A_2A_R-KO mice, suggesting that the effects of A_2A_R on wound healing are mainly mediated through endothelial cells. However, the mechanism by which A_2A_R regulates wound healing has not been established and is likely multifactorial, as it affects inflammation^15^, ^16^, fibroblasts and epithelial cells 13, 17, 18. On the one hand, these findings regarding the effects on fibroblasts and epithelial cells are based on the use of agonists and *in vitro* experiments^2^, ^17^, ^72^, ^73^, and there are specific concerns about the effects of agonists and their widespread regulation compared with those of exogenous substances. Moreover, regarding the effect of A_2A_R on fibroblasts, researchers have focused mainly on its role in fibrotic diseases^71^, ^74^. On the other hand, for the regulation of inflammatory cells, an induced deficiency in A_2A_R is not associated with a defect in the acute inflammatory response or with a difference in the number or functional capacity of A_2A_R-deficient leukocytes^15^, and convincing *in vivo* experimental evidence for a role of A_2A_R in wound healing through inflammatory reactions is lacking^75^. In addition, some research has shown that A_2A_R regulates inflammation through ECs^76^. Thus, A_2A_R's effects on ECs play a critical role in the regulation of wound healing. However, further studies are needed to determine the role of A_2A_R in other cells during wound healing.

Interestingly, we also found that the deficit in granulation tissue was obvious at 6 and 9 days postwounding, whereas the extent of re-epithelialization did not differ between the WT controls and the A_2A_R KO mice, which is consistent with the findings of a previous experiment^15^, ^54^. Moreover, EC-A_2A_R KO mice presented similar results and were not significantly different from A_2A_R KO mice, strongly suggesting that A_2A_R regulates granulation tissue formation, but not epithelialization, during wound healing. Previous studies have indirectly demonstrated that A_2A_ R is involved in the angiogenic effects of ECs by total A_2A_R KO or the application of agonists or antagonists^13^, ^15^, ^51^, which critically affects the formation of granulation tissue, and these results are consistent with the observed defects in granulation tissue formation. Importantly, our study directly demonstrated that A_2A_R affects granulation tissue formation mainly by regulating angiogenesis by using EC-A_2A_R KO mice, further indicating that the angiogenic effects of A_2A_R on ECs play a critical role in the regulation of wound healing. Similarly, Liu *et al.* demonstrated that A_2A_R is crucial for pathological angiogenesis in proliferative retinopathies using EC-A_2A_R-KO mice^57^.

Angiogenesis promotes growth, development, and wound healing through the formation of granulation tissue^77^. Among the many cells involved in the angiogenic process, ECs play an essential role in new vessel formation via an increase in cell proliferation, migration, and tube formation capacity^78^. Although cell proliferation, migration, and tube formation capacity were assessed *in vitro* in this study, only the number of CD31-positive cells was determined *in vivo*. In future experiments, we should use other parameters to clarify this process *in vivo*. In addition to the above effects, further exploration is needed to determine whether A_2A_R regulates angiogenesis through other mechanisms or can regulate angiogenesis by influencing other cellular effects (for example, inflammatory cells).

However, previous reports have shown that the angiogenic effect of A_2A_R activation is likely mediated by increased expression of VEGF in ECs^79^-^81^. Other studies have also reported that A_2A_R stimulation increases angiogenesis through the antiangiogenic matrix proteins thrombospondin 1^64^, annexin A2^56^, and MyD88^16^ and macrophage exosomes^55^.

We demonstrated that A_2A_R regulates angiogenesis through c-Ski. c-Ski was significantly expressed on ECs during wound healing and was significantly decreased after A_2A_R KO and EC-A_2A_R KO. A_2A_R activation induced a proangiogenic effect and increased the expression of c-Ski, whereas c-Ski depletion abolished the proangiogenic effects of A_2A_R activation. In addition, similar to our previous research^26^, ^82^, the expression of c-Ski increased after wounding, peaked at 6 days and then significantly decreased, which is consistent with the changes in A_2A_R expression after wounding. Furthermore, c-Ski is expressed on ECs^30^, and reduced c-Ski expression in the wound results in significantly slower wound healing^26^, similar to the results of A_2A_ R KO. Taken together, these results strongly indicate that c-Ski plays an essential role in A_2A_R-mediated angiogenesis. More importantly, c-Ski depletion reduced the proliferation and tube formation of HMECs and abolished the promotive effects on cell proliferation and tube formation caused by A_2A_R activation *in vitro*, further demonstrating the important role of c-Ski in A_2A_R-mediated angiogenesis.

Although there is little research on the role of c-Ski in angiogenesis or EC regulation, as a multieffector factor, this molecule can regulate the proliferation and migration of fibroblasts^83^, vascular smooth muscle cells^84^, cardiomyocytes^85^, astrocytes^86^, and tumor cells^87^, ^88^. These results indicate that c-Ski may regulate angiogenesis through these effects. Moreover, the overexpression of c-Ski was shown to promote the proliferation of ECs^31^, ^32^. In addition, an analysis of the regulated signal transduction pathway revealed that c-Ski not only synergizes with factors such as activator protein-1 (AP-1)^89^ and signal transducer and activator of transcription 3 (STAT-3)^90^, which upregulate the expression of the essential angiogenic factor VEGF^91^, ^92^, but can also reduce the expression of the antiangiogenic factor thrombospondin-1 (TSP-1)^93^, suggesting that c-Ski plays an important role in angiogenesis. Interestingly, A_2A_R can regulate angiogenesis not only through VEGF^18^,^19^,^20^ but also through TSP1^64^, further indicating that A_2A_R can regulate angiogenesis through c-Ski. Nevertheless, the mechanism of the proangiogenic effect of c-Ski remains to be further explored.

As reported in previous studies, the ERK/CREB pathway is an important pathway for regulating c-Ski expression^40^. Our study revealed that MEK inhibition markedly reduced c-Ski expression and abrogated A_2A_R-mediated promotion of c-Ski expression with CGS21680 in HMECs, indicating that A_2A_R regulates c-Ski protein expression in HMECs through the ERK1/2 pathway. Our results demonstrated that c-Ski expression is regulated by A_2A_R in ECs through this pathway, and the results of other studies support this finding. Several studies have demonstrated that the ERK pathway is important in angiogenesis^94^, ^95^ and A_2A_R activation can activate the ERK pathway in ECs^52^, ^96^-^98^. In particular, Liu *et al.* reported that A_2A_R activation promotes pathological angiogenesis via ERK-dependent translational activation^57^. In addition, our study revealed that A_2A_R activation by CGS21680 enhanced the ERK1/2 pathway through PKA, which is consistent with the findings of several previous reports^99^, ^100^. However, further *in vivo* experimental verification of A_2A_R activation inducing the expression of c-Ski through the ERK/CREB pathway is needed.

## Conclusions

Taken together, our results demonstrate that the delayed effects of A_2A_R KO on wound healing are due mainly to the effects of A_2A_R on endothelial cells, indicating that A_2A_R-mediated angiogenesis plays a critical role in wound healing. Moreover, our results demonstrate a novel cellular and molecular mechanism whereby endothelial A_2A_R deficiency delays wound healing and decreases angiogenesis through c-Ski deficiency. A_2A_R regulates the expression of the c-Ski protein through the ERK/CREB pathway in HMECs. Thus, these findings provide new insights into a previously unrecognized effect of A_2A_R on angiogenesis by ECs in wounds and highlight the mechanism of A_2A_R in wound repair, which not only identifies A_2A_R as a therapeutic target in wound repair but also highlights other angiogenesis-dependent tissue repair processes.

## Supplementary Material

Supplementary figures.

## Abbreviations

A_2A_R: adenosine A_2A_ receptor
ECs: endothelial cells
c-Ski: Cellular Sloan-Kettering Institute
A_2A_R KO: A_2A_R knockout
EC-A_2A_R KO: EC-specific A_2A_R knockout
HMECs: human microvascular ECs
ERK1/2: signal-regulated kinase 1/2
WT: wild-type
H&E: hematoxylin and eosin
CGS21680: 2-p-[2-carboxyethyl]phenethyl-amino-5'-N-ethylcarboxamido-adenosine
DMSO: dimethyl sulfoxide
AP-1: activator protein-1
STAT-3: signal transducer and activator of transcription 3
TSP-1: thrombospondin-1
FBS: fetal bovine serum
PBS: phosphate-buffered saline
IntDen: integrated density
STR: short tandem repeat
HBSS: Hank's balanced salt solution
OD: optical density
