# Supplementary material for: The Essential Role of Angiogenesis in Adenosine 2A Receptor Deficiency-mediated Impairment of Wound Healing Involving c-Ski via the ERK/CREB Pathways
Source: Int J Biol Sci. 2024 Aug 19;20(11):4532–50. doi: 10.7150/ijbs.98856 (PMC11380447; doi:10.7150/ijbs.98856)
Supplement: Supplementary file 1 — Supplementary figures. [file ijbsv20p4532s1.pdf]

Supplemental fig. 1

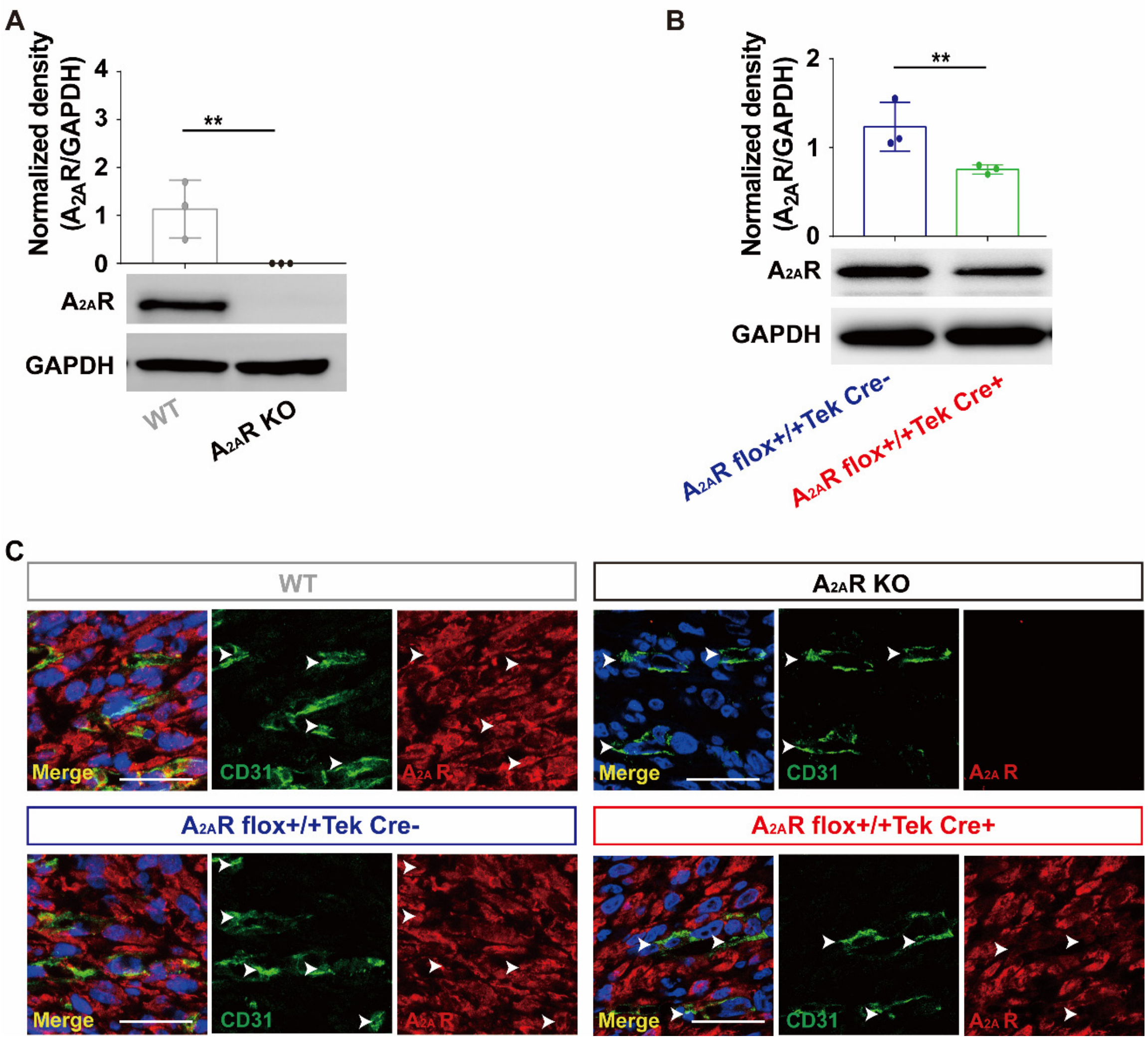

Figure S1. The expression of A_{2A}R in A_{2A}R KO and EC-A_{2A}R KO mice.

Western blot analysis of A_{2A}R protein expression in full-thickness skin in A_{2A}R KO (A) and EC-A_{2A}R KO mice (B). ***$p < 0.01$ (n=3). (C) Double-label immunofluorescence for A_{2A}R (red) and CD31 (green) in the granulation tissue of the wound at 9 days post-wounding revealed the expression of A_{2A}R in CD31-positive cells and nonpositive cells. Scale bar, 50 µm.

Supplemental fig. 2

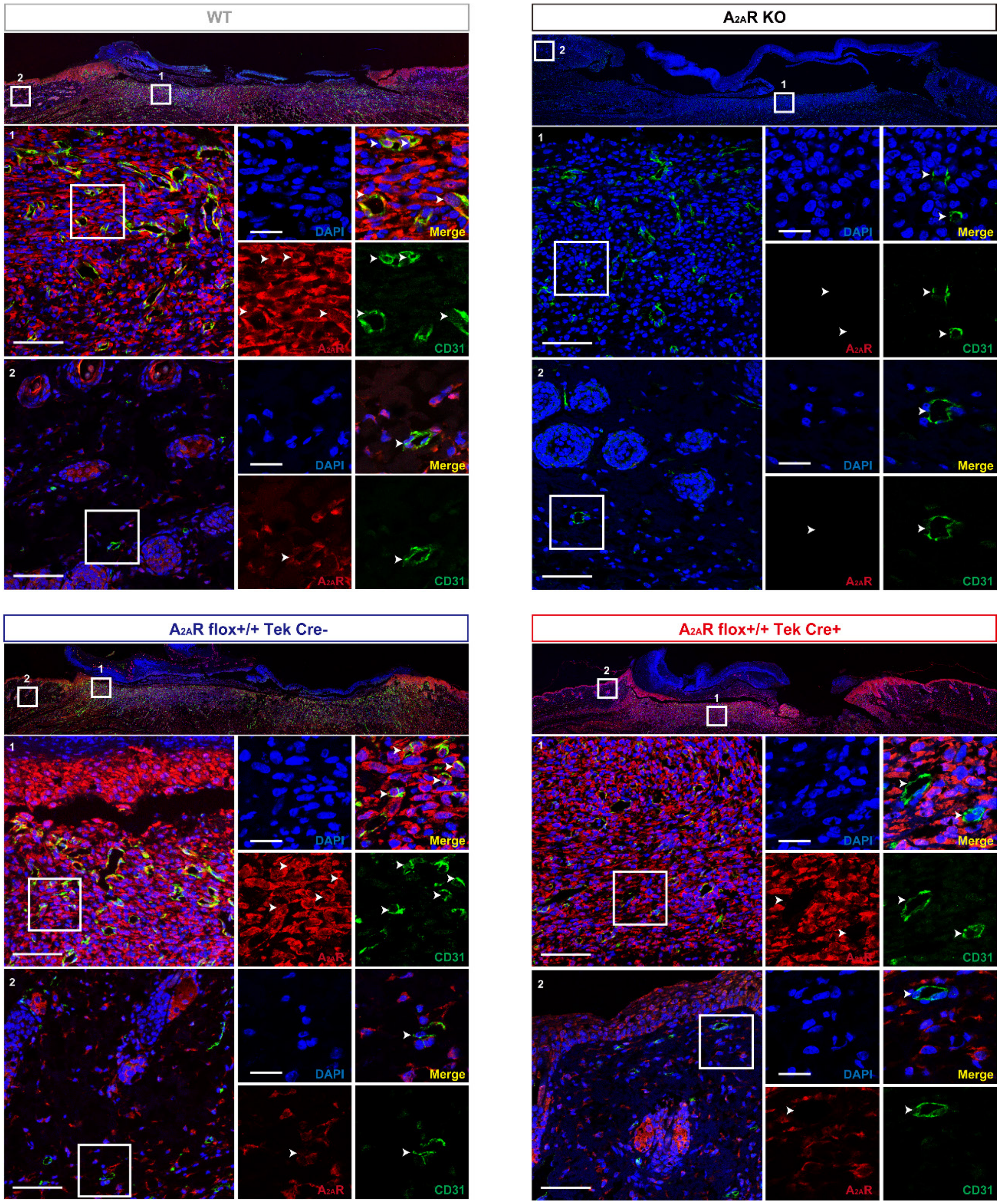

Figure S2. The expression and colocalization of $A_{2A}R$ and CD31 in $A_{2A}R$ KO and EC-$A_{2A}R$ KO mice after full-thickness wounding.

Double-label immunofluorescence for $A_{2A}R$ (red) and CD31 (green) in undamaged subcutaneous tissue and the granulation tissue of the wound at 9 days post-wounding revealed the colocalization of $A_{2A}R$ and CD31 (white arrow). Short scale bar, 100 µm; Long scale bar, 50 µm.

Supplemental fig. 3

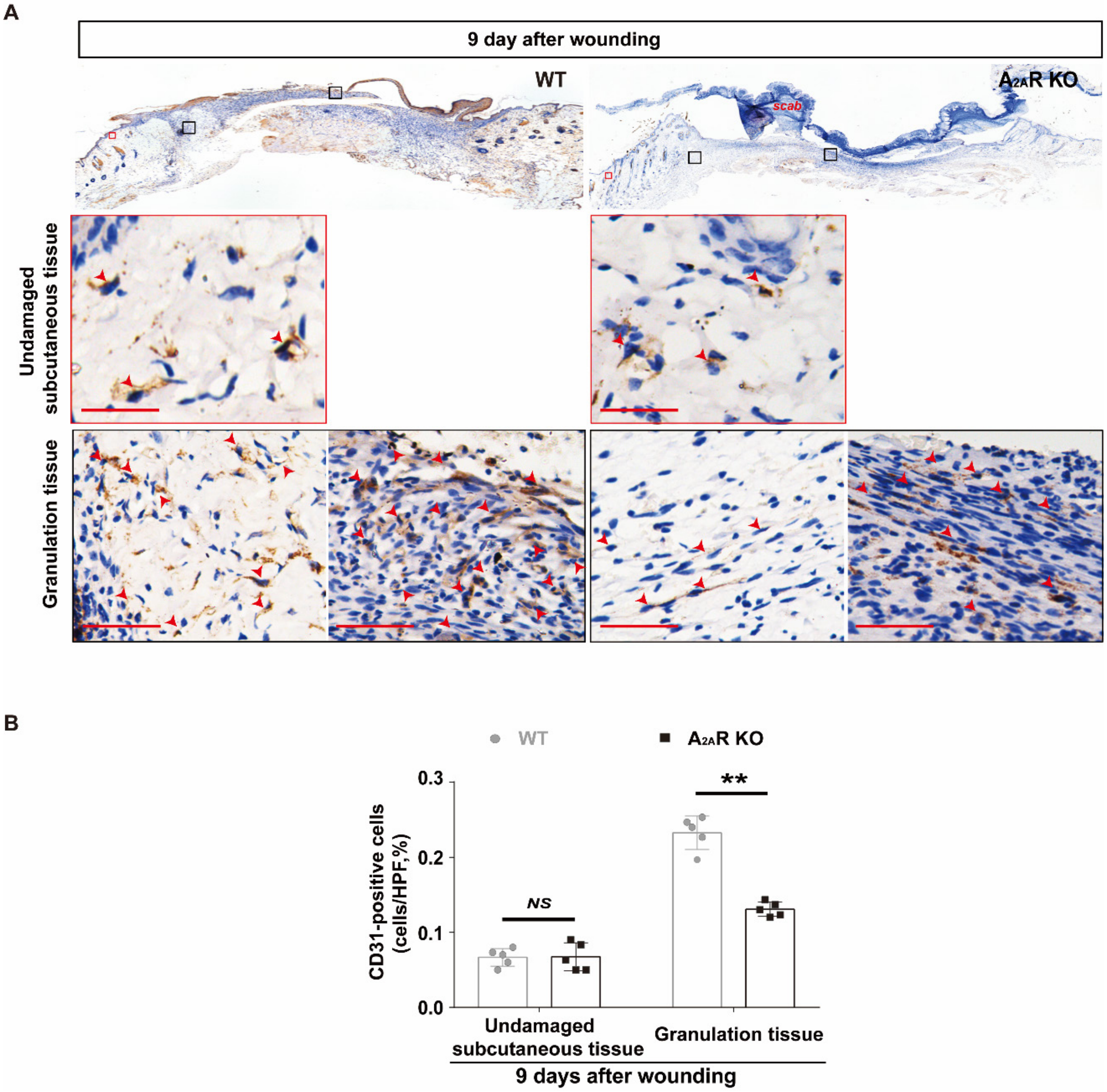

Figure S3. Changes in angiogenesis in $A_{2A}R$ KO mice after full-thickness wounding. (A) Immunohistochemistry for CD31 (red arrow) in $A_{2A}R$ KO mice at 9 days post-wounding. Scale bar, 50 µm. (B) Quantitative analysis of CD31-positive cells in each group. $**p < 0.01$ (n = 5); NS, not significant.

Supplemental fig. 4

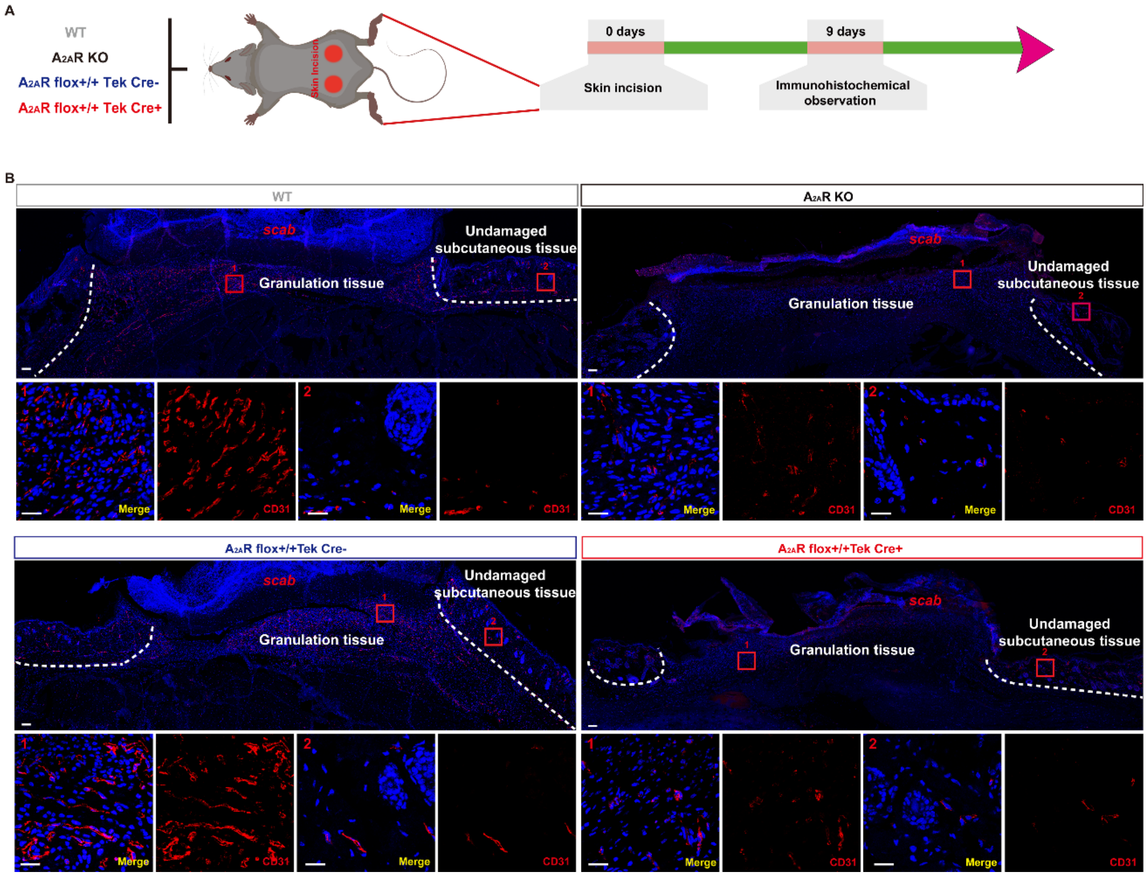

Figure S4. Changes in angiogenesis in $A_{2A}R$ KO and EC-$A_{2A}R$ KO mice after full-thickness wounding.

(A) Experimental procedure. (B) Immunohistochemistry for CD31 (red arrow) in $A_{2A}R$ KO and EC-$A_{2A}R$ KO mice at 9 days post-wounding. The lower images are higher magnification sections of the red squares in the upper images. Short scale bar, 200 µm; Long scale bar, 50 µm.

Supplemental fig. 5

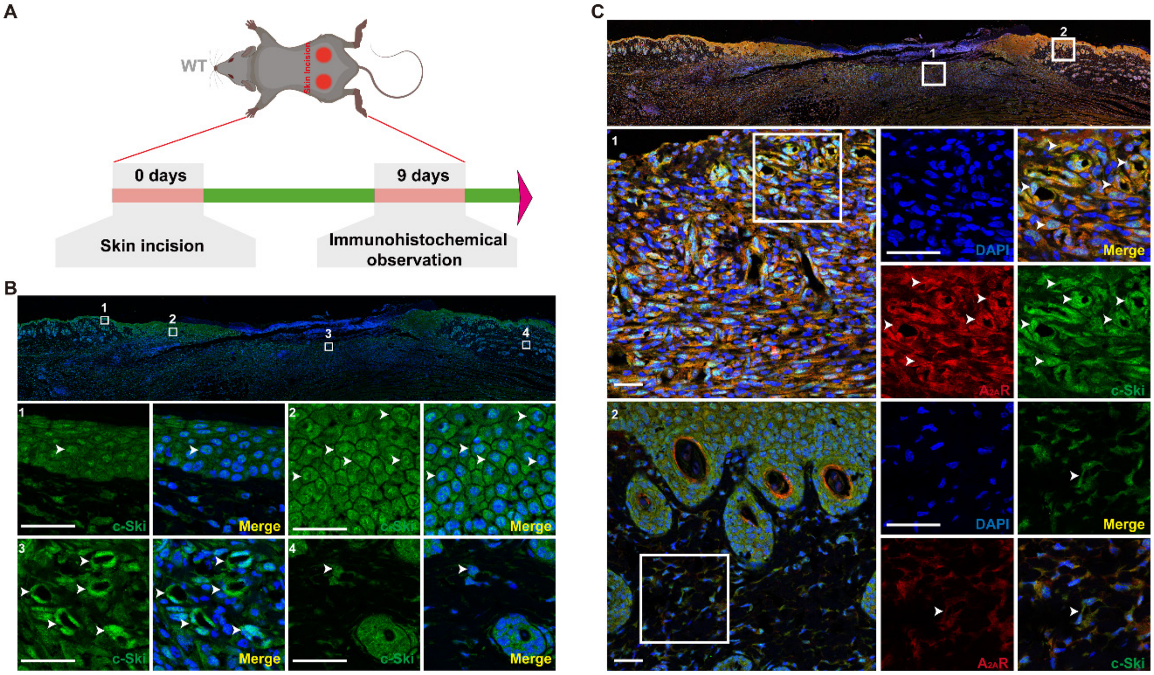

Figure S5. Colocalization of c-Ski and $A_{2A}R$ in the granulation tissue of WT mice after full-thickness wounding.

(A) Experimental procedure. (B) The expression of c-Ski (green, white arrow) in the epithelium, neoepithelium, undamaged subcutaneous tissue and granulation tissue at 9 days post-wounding in WT mice. Short scale bar, 50 µm; Long scale bar, 50 µm. (C) Double-label immunofluorescence for c-Ski (green) and $A_{2A}R$ (red) in undamaged subcutaneous tissue and the granulation tissue of the wound at 9 days post-wounding revealed the colocalization of c-Ski and $A_{2A}R$ (white arrow). Short scale bar, 50 µm; Long scale bar, 50 µm.

Supplemental fig. 6

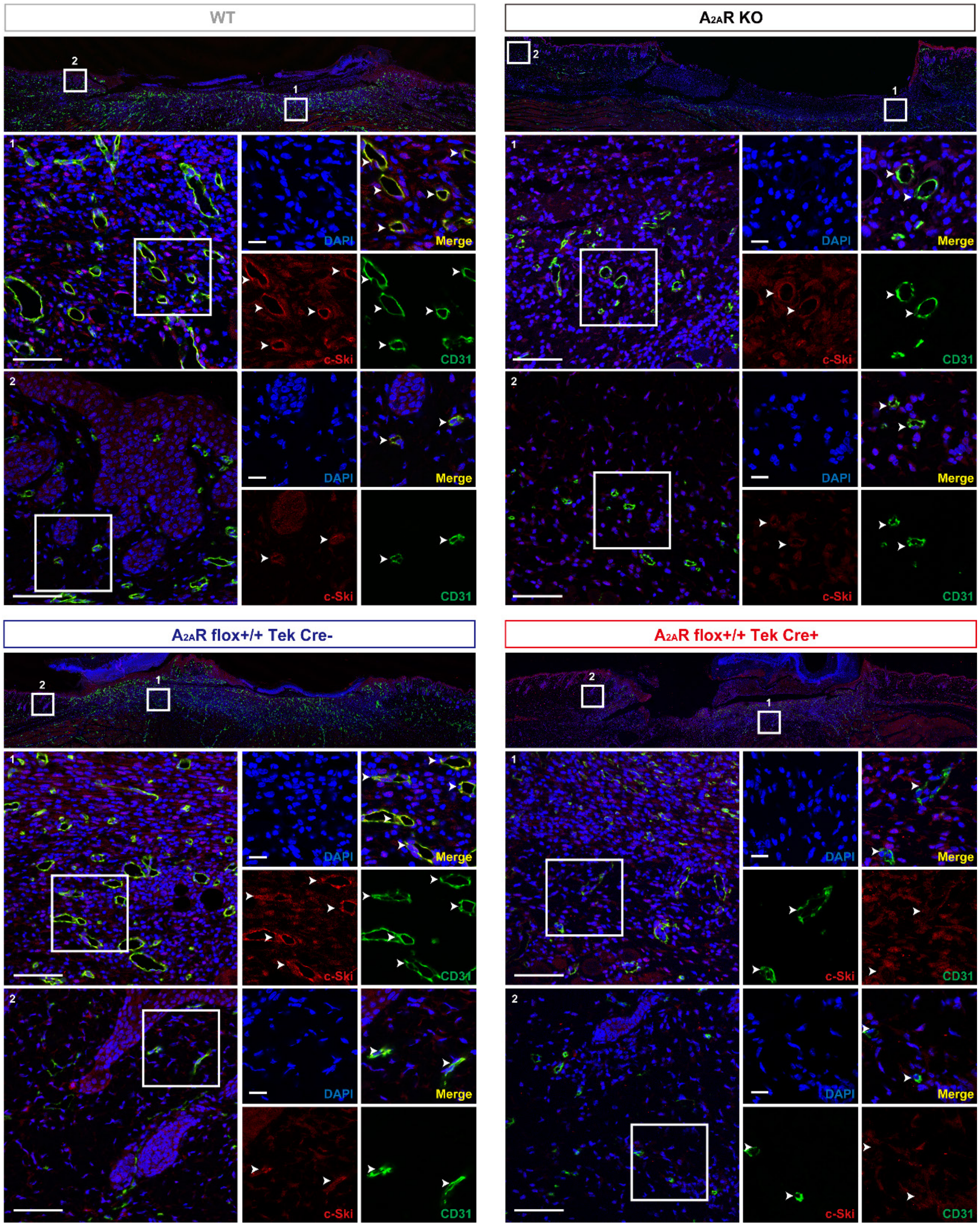

Figure S6. The expression and colocalization of c-Ski and CD31 in $A_{2A}R$ KO and EC-$A_{2A}R$ KO mice after full-thickness wounding.

Double-label immunofluorescence for c-Ski (red) and CD31 (green) in the undamaged subcutaneous tissue and the granulation tissue of the wound at 9 days post-wounding revealed the colocalization of c-Ski and CD31 (white arrow). Short scale bar, 50 μm; Long scale bar, 50 μm.

Supplemental fig. 7

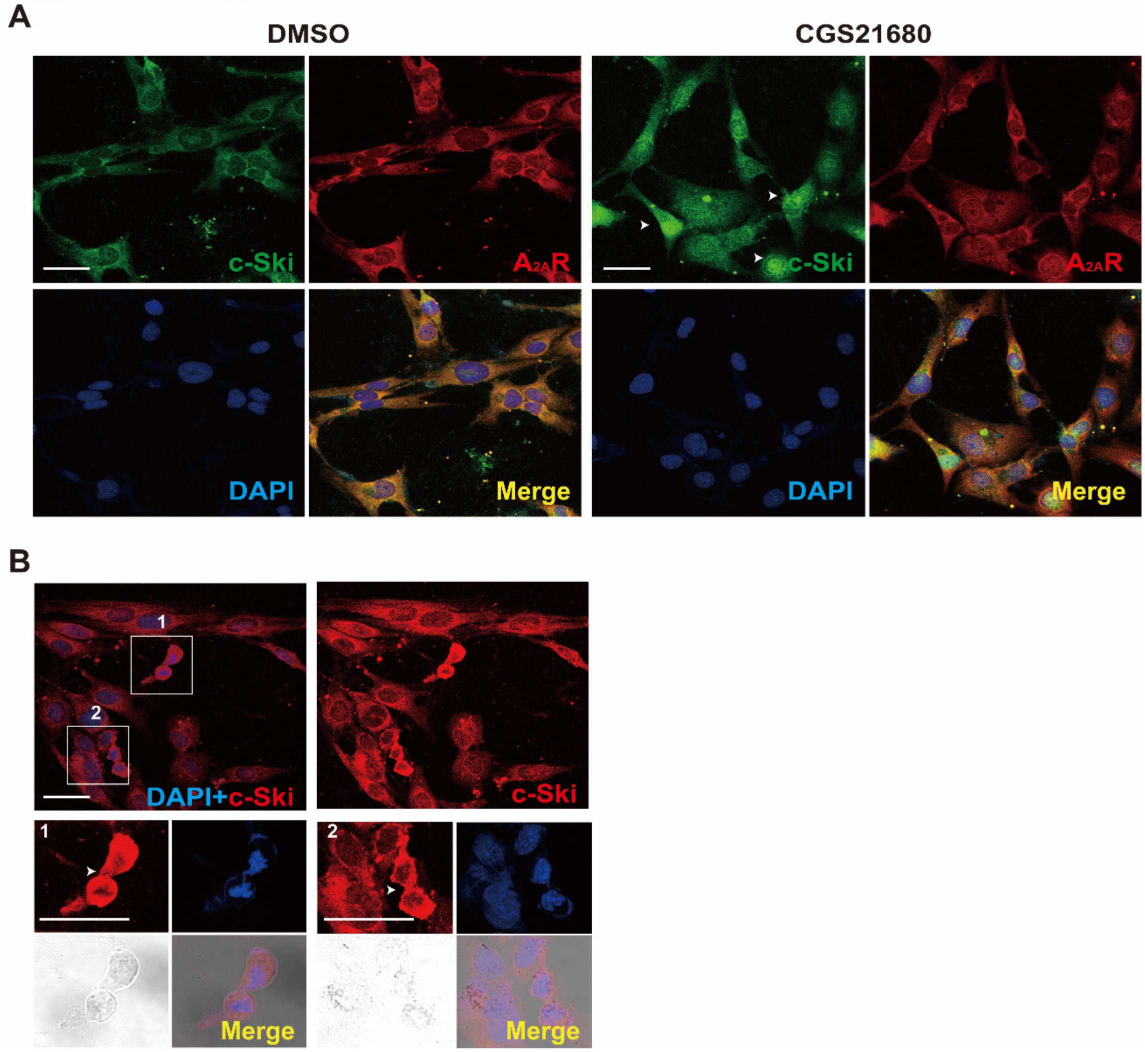

Figure S7. Expression of c-Ski and $A_{2A}R$ in HMECs after CGS-21680 treatment.

(A) Double-label immunofluorescence for c-Ski (green) and A_2AR (red) in HMECs at 12 h after CGS21680 treatment revealed increasing levels of c-Ski and obvious nuclear aggregation (white arrow). Scale bar, 50 µm. (B) The fluorescence intensity of c-Ski (red) obviously increased in dividing cells (white arrow). The lower panel shows higher magnification sections of the white squares in the upper panel. Short scale bar, 50 µm; Long scale bar, 50 µm.

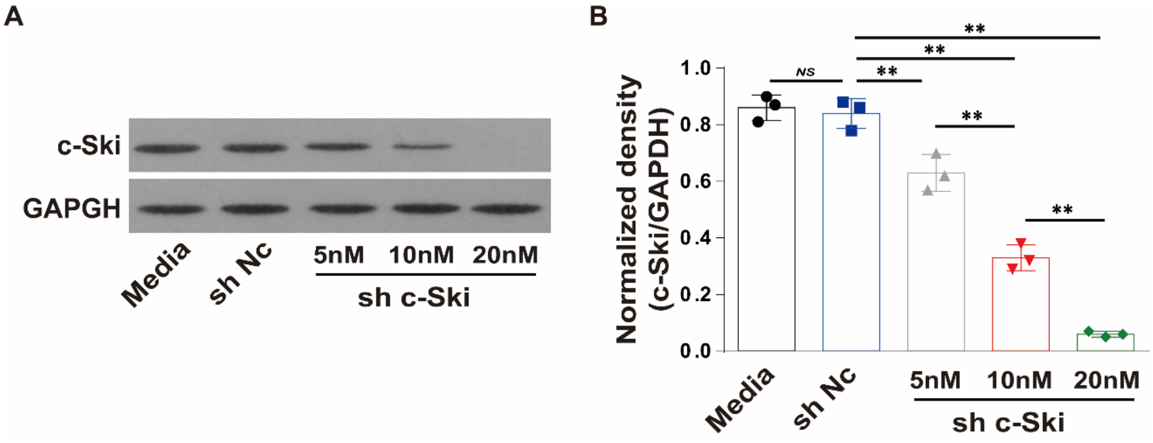

Figure S8: WB analysis of c-Ski protein levels in HMEC-1 cells. HMEC-1 cells were treated with 5 nM, 10 nM, or 20 nM sh c-Ski for 24 h. Representative images (A) and western blot (B) analysis of c-Ski protein levels in U937 cells. The results were obtained from three independent experiments. $^{**}p < 0.01$ (n = 3); NS, not significant.

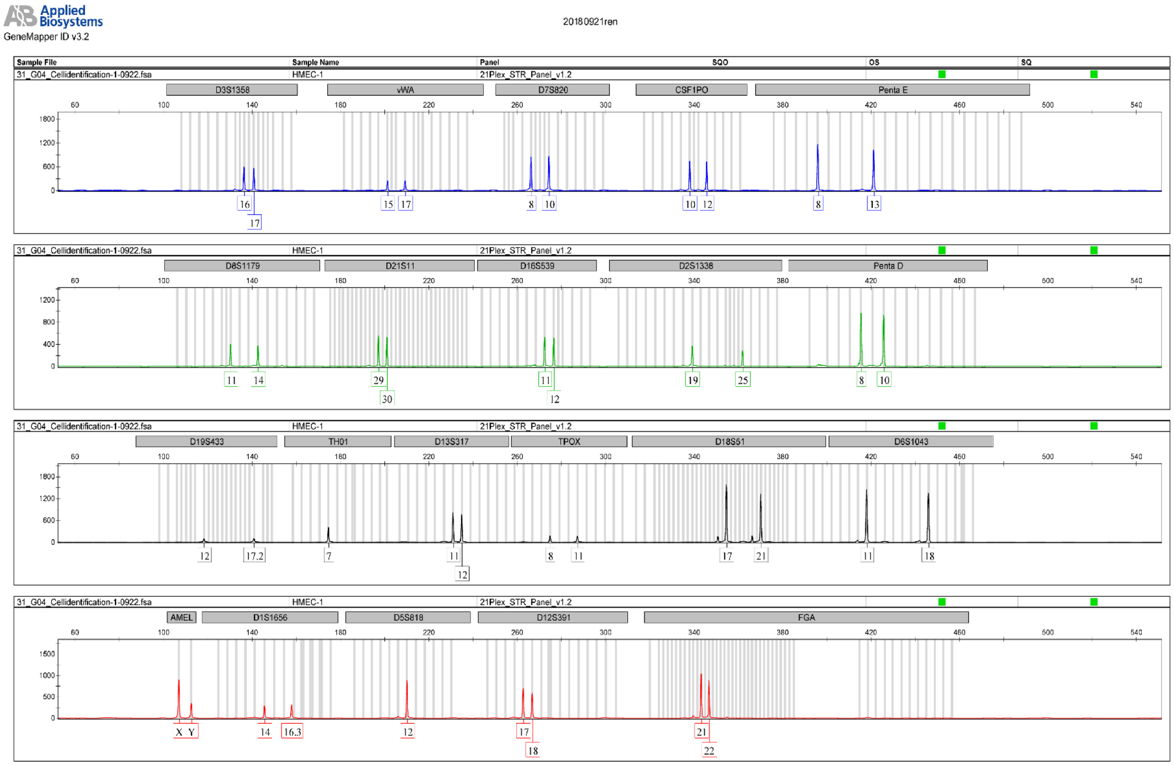

Figure S9: STR profiles of sample cell line

The submitted profile is exact match for the following human cell line(s) in the DSMZ STR database (8 core loci plus Amelogenin): HMEC-1.
